# App-based mindfulness meditation training enhances cognitive flexibility and modulates ACC and medial frontal gyrus activation during task switching in adolescent OCD

**DOI:** 10.1016/j.ynirp.2026.100347

**Published:** 2026-04-23

**Authors:** Sarah Rempel, Maria McDonald, Veit Roessner, Christian Beste, Nicole Beyer

**Affiliations:** a Department of Child and Adolescent Psychiatry, Faculty of Medicine of the TU Dresden, 01307, Dresden, Germany; b Cognitive Neurophysiology, Department of Child and Adolescent Psychiatry, Faculty of Medicine of the TU Dresden, 01307, Dresden, Germany; c German Center for Child and Adolescent Health (DZKJ), partner site Leipzig/Dresden, Dresden, Germany

**Keywords:** Cognitive flexibility, OCD, Mindfulness, Task switching, MVPA, EEG

## Abstract

**Background:**

Cognitive flexibility is a core executive function often impaired in individuals with obsessive-compulsive disorder (OCD). Mindfulness-based interventions have been hypothesized to improve cognitive flexibility and may be a promising adjunctive intervention for adolescents with OCD. The current study examines which cognitive neurophysiological processes underlie mindfulness-based interventions in cognitive flexibility.

**Methods:**

In a randomized, interviewer-blind design, 76 adolescents (aged 10-19) with diagnosed or suspected OCD were enrolled. After exclusion, the final sample included 53 participants (45.3 % male). Participants were randomly assigned to either a mindfulness training group or an active control group (audiobook listening). Cognitive flexibility was assessed using a task-switching paradigm while EEG data were collected. Multivariate pattern analysis (MVPA) and source localization were applied to decomposed EEG data, enabling control of inter-individual variability.

**Results:**

The mindfulness group showed significantly reduced switch costs compared to the control group, indicating improved cognitive flexibility. The MVPA revealed group-specific decoding performance effects in fractions of EEG data reflecting response selection processes in a time window of 300-400 ms. In this time frame, the mindfulness group showed differential activation in the medial frontal gyrus and anterior cingulate cortex.

**Conclusions:**

This study provides evidence that an app-based mindfulness meditation training can enhance cognitive flexibility in adolescents with OCD through modulating stimulus-response selection processes in frontal cortices.

## 1 Introduction

Cognitive flexibility is the ability to switch between different mental tasks or strategies based on changing environmental demands and is considered an essential aspect of executive functions (EF) (Diamond, 2013). Changes in cognitive flexibility are evident in obsessive-compulsive disorder (OCD), a disorder affecting 1-2% of children and adolescents. Individuals with OCD often exhibit impairments in situations that require task switching (Abramovitch et al., 2013; Britton et al., 2010; Chamberlain et al., 2007, 2021; Gruner and Pittenger, 2017; Gu et al., 2007; Shin et al., 2014; Snyder et al., 2015; Wolff et al., 2017a). Difficulties in cognitive flexibility in OCD may lead to perseveration on thoughts or behaviors, which can exacerbate the disorder's symptoms (Gruner and Pittenger, 2017; Snyder et al., 2015). Research has shown that during task switching, individuals with OCD show different cortical activation patterns (Britton et al., 2010; Gruner and Pittenger, 2017; Remijnse et al., 2013) compared to healthy controls, and other evidence reveals that this may play a role in the development and maintenance of the disorder (Gruner and Pittenger, 2017; Gu et al., 2007; Remijnse et al., 2013; Wolff et al., 2017a). So far, no single model can provide a complete explanation for the role of cognitive flexibility in OCD, as there are likely to be interactions between different brain areas (Bragdon et al., 2022) as well as interactions between cognitive flexibility and other EF (Wolff et al., 2017a, 2018). This highlights the importance of investigating cognitive flexibility in OCD and its potential implications for interventions with a differential neurophysiological view. Cognitive flexibility can be examined in task switching paradigms, where the inhibition of the mental representation of the previous task is needed to switch to a new task (Diamond, 2013; Kiesel et al., 2010). Generally, responses to switch trials take longer to process than responses to repeat trials. This phenomenon is known as switch costs, where smaller switch costs indicate better cognitive flexibility (Monsell, 2003). To better understand the neural mechanisms underlying such cognitive flexibility, more sophisticated neurophysiological approaches are required.

First, we applied the method Residue Iteration Decomposition (RIDE) to the single-trial EEG data. RIDE is conceptually important because it separates overlapping cognitive processes into functionally distinct clusters, providing a more precise temporal mapping (Chmielewski et al., 2017; Ouyang et al., 2017; Petruo et al., 2021). It assumes that ERPs are composed of multiple components with different latencies, reflecting different stages of cognitive processing. Specifically, RIDE decomposes the EEG into three clusters: the S-cluster, reflecting stimulus-related processes; the C-cluster, capturing intermediate processes between stimulus evaluation and response selection; and the R-cluster, reflecting response-related processes related to movement execution (Ouyang et al., 2011, 2015). Traditional univariate analyses of EEG data (e.g., event-related potentials [ERPs]) are limited in their ability to capture how neural representations evolve over time, as they do not assess the temporal generalization of decodable information across different time points (Hebart and Baker, 2018; King and Dehaene, 2014; Takács et al., 2022a). To overcome these limitations, several non-traditional EEG analysis approaches integrate multiple measures to capture complex neural dynamics. For example, time-frequency analyses combine information across frequencies and time (Morales and Bowers, 2022), functional connectivity measures assess interactions between brain regions over time (Chiarion et al., 2023), and EEG microstate analysis considers both spatial topographies and their temporal sequence (Michel and Koenig, 2018). While these methods capture multiple aspects of the signal, they do not directly test whether distributed EEG patterns encode task-specific information. In contrast, MVPA explicitly evaluates whether multivariate EEG patterns discriminate between experimental conditions (i.e., switch vs. repetition trials) and how such representations evolve across time (Fahrenfort et al., 2018; Grootswagers et al., 2017; King and Dehaene, 2014; Petruo et al., 2021). In our study, group-level decoding assessed condition-specific information at each time point, while temporal generalization examined the stability and generalizability of these representations across time. To further disentangle overlapping cognitive processes, we applied MVPA to RIDE-decomposed clusters (Petruo et al., 2021). Thus, MVPA combined with RIDE decomposition provides a powerful approach to track the temporal dynamics of cognitive flexibility during task switching.

Based on previous literature, mindfulness meditation was hypothesized to reduce switch costs (Chambers et al., 2008; Hartkamp and Thornton, 2017). Mindfulness is a type of awareness that arises from the non-judgmental observation of the present moment (Kabat-Zinn, 2003). By facilitating non-judgmental awareness of unwanted and intrusive thoughts and reducing the importance attached to these thoughts, mindfulness can help individuals with OCD to relieve their symptoms (Gu et al., 2015; Segal et al., 2018). Rather than attempting to suppress thoughts, mindfulness-based approaches encourage individuals with OCD to acknowledge and accept intrusive urges (Külz et al., 2019; Lee et al., 2022). Mindfulness-based interventions like meditation may reflect an effective adjunct treatment for patients with OCD, as supported by meta-analyses in adults (Bürkle et al., 2025; Chien et al., 2022; Lee et al., 2022; Riquelme-Marín et al., 2022). To our knowledge, no studies have yet investigated mindfulness-based interventions in children or adolescents with OCD.

This study investigated the effects of an app-based mindfulness meditation training on cognitive flexibility in adolescents with OCD. Based on previous literature, we hypothesized that behaviorally, the mindfulness intervention group would show improved cognitive flexibility, reflected in lower switch costs (Hartkamp and Thornton, 2017), compared to the audiobook control group, for which no changes in cognitive flexibility were expected. We also expected to see neurophysiological effects in the EEG data using MVPA on the RIDE-decomposed clusters in the mindfulness intervention group in the group-level decoding (between switch vs. repetition). Specifically, we expected to see the impact in the C-Cluster, since recent data, applying RIDE to the same task switching paradigm (in adult participants without OCD), also found effects in the C-Cluster (Wolff et al., 2017b). These were attributed to mechanisms related to updating internal representations. Although some studies have shed light on the neural mechanisms underlying cognitive flexibility in OCD during task switching (Gruner and Pittenger, 2017; Wolff et al., 2017a), there is a lack of studies with children and adolescents, interventions, and MVPA. Additionally, we explored whether an app-based mindfulness training might also influence clinical symptoms by assessing OCD severity using the Children's Yale-Brown Obsessive Compulsive Scale (CY-BOCS), a widely used measure of obsessions and compulsions in children and adolescents (Scahill et al., 1997).

## 2 Methods and materials

### 2.1 Participants

In this randomized and interviewer-blind study, we enrolled N = 76 adolescents aged between 10 and 19 years who had been diagnosed with OCD according to ICD-10 criteria or had suspected OCD with a CY-BOCS score of at least 8 points. Exclusion criteria were treatment for other primary psychiatric disorders, neurological or developmental disorders, a history of psychosis, a current severe depressive episode, current substance use disorder, acute suicidal tendencies, or an IQ less than 70 (administered by “Der Zahlen-Verbindungs-Test” [ZVT] (Oswald and Roth, 1987)). Additionally, participants were not allowed to change their existing therapy during the study (e.g., medical, behavioral therapy, etc.). 23 participants were excluded from the analyses due to outliers in the behavioral data of the task switching paradigm, technical difficulties during EEG measurement, exclusion after pre-processing the EEG data, low app compliance, appointment issues, and dropouts (c.f. Table A1). The final sample consisted of 53 adolescents (M_age_ = 15.4 years ± 2.08; M_IQ_ 102.74 ± 13.96; 45.3 % male). All participants had normal or corrected-to-normal vision. Possible comorbidities were assessed using a semi-structured interview (Kinder-DIPS (Schneider et al., 2017), based on DSM-5 criteria and current clinical reports, with N = 18 of the final sample having comorbid diagnoses and N = 17 taking medication (c.f. Table A2).

### 2.2 Procedure

The study was conducted in accordance with the Declaration of Helsinki, and approved by the Ethics Committee of TU Dresden (protocol code EK18012019 on 25 April 2019). Informed consent was obtained from both parents and participants prior to participation. Participants were recruited from the department of child and adolescent psychiatry and psychotherapy at the university hospital Dresden, Germany. They were randomly assigned to either a mindfulness group (using an app-based mindfulness meditation training) or an audiobook group (active control group). To maintain the study blind, all participants were assigned a re-identified study ID. All participants underwent two EEG recording appointments (pre-appointment and post-appointment, following the 8-week application), during which they performed the task switching paradigm and a backward inhibition (BI)-paradigm in randomized order. The results of the BI-paradigm can be found in a published article (Rempel et al., 2023). The mindfulness group used the app “7Mind”, which contained several meditation courses (cf. Intervention). In contrast, the audiobook group used the app “Audible” with the audiobooks “Harry Potter and the Philosopher's Stone” and “Harry Potter and the Chamber of Secrets”. Both groups used their respective apps on a smartphone for 8 weeks, twice a day, following a structured plan provided to them at their first appointment. Participants were asked to maintain compliance by staying in contact with the study team via a smartphone messenger service and had an interim appointment after four weeks to discuss progress.

### 2.3 Intervention

The mindfulness group received the app ‘7Mind’ with the following meditation courses: Kids & school (Kids & Schule), Basics (Grundlagen), Basics intensive 1 (Grundlagen Intensiv 1), Basics intensive 2 (Grundlagen Intensiv 2), Basics intensive 3 (Grundlagen Intensiv 3), Basics intensive 4 (Grundlagen Intensiv 4), luck (Glück), gratitude (Dankbarkeit), sleep (Schlaf). The app can be downloaded in the Google play store or App store (https://www.7mind.de/download). Each audio mindfulness meditation course contains seven sessions of seven – 12 min each. Participants were asked to practice one mindfulness meditation during the day and one when going to bed (sleep meditation) as outlined by the structured intervention plan participants received. A calm, male voice gave instructions, followed the same structure, and thus made it easier for the participant to internalize the dynamics of the meditation process more and more deeply. The audiobook group used the app Audible (https://www.audible.de/ep/apps) with the audiobooks “Harry Potter and the Philosophers Stone” for the first 4 weeks and “Harry Potter and the Chamber of Secrets” for the following 4 weeks, as (also) outlined by the structured intervention plan participants received. Both groups used the app on a smartphone for 8 weeks, twice a day.

### 2.4 Task switching paradigm

Participants were seated in front of a computer screen and performed a task-switching paradigm (Gajewski et al., 2011; Wolff et al., 2017a). We used "Presentation" software (version 14.9) for stimulus presentation, response recording, and EEG triggers. During the task, participants sat in front of a computer monitor with a viewing distance of approximately 60 cm in the EEG-laboratory of the Department of Child and Adolescent Psychiatry of the University Hospital Dresden. Written instructions were displayed on the screen and verbally explained. Participants were encouraged to respond as quickly and accurately as possible. A short practice block with 12 trials was completed before both the pre- and post-appointment to ensure comprehension.

**Fig. 1:**
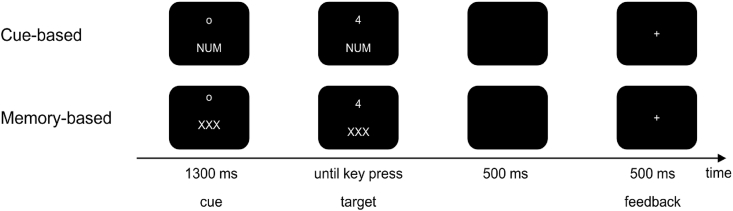
The outline of the task switching paradigm. The cue-based task switching paradigm is shown in the upper part, the memory-based in the lower part of the figure. In the memory-based part, the participants had to remember when to switch a rule.

Participants had to determine whether the presented number was greater or less than 5 in the numeric rule, odd or even in the parity rule, and small or large in the font-size rule indicated by cues (Fig. 1). The stimuli were numbers 1-9 (excluding 5), presented in white font on a black screen in either small or large size.

Trials were structured as follows: first, a fixation point in combination with the cue stimulus was presented for 1300 ms (ms). The cue-stimulus remained visible until the digit (target) was presented. The digit appeared at the same position at which the fixation point was presented before. A response had to be given within 2500 ms after target-onset. If no response was given within the 2500 ms, the trial was rated as missed response. 500 ms after the response, feedback was displayed for 500 ms, which was either a plus (in case of a correct response) or a minus sign (after a wrong response). After the feedback the next cue was shown. The response-cue interval (RCI) was set to ∼1500 ms and included the response-feedback delay (500 ms), the feedback (500 ms) and the feedback-cue delay jittered between 400 and 600 ms (mean 500 ms). The paradigm comprised 768 trials, divided into eight blocks (4 cue-based blocks, four memory-based blocks). Depending on the cue, they responded via button presses with their left or right index finger. The cues were “GER” (rule odd or even = rule A), a “NUM” (rule smaller or larger than 5 = rule B), or “SG” (small font size 50 vs. large font size 80 = rule C). During the cue-based block the rules alternated randomly with an equal probability of cue occurrence and a frequency of task switching of 50%. During the memory-based block, participants were told to switch the rule after every third trial out of memory: starting with the numeric task, which needed to be repeated three times. Afterwards, the parity task and then the font-size task were valid, with each task needing to be repeated three times. Then, participants were required to start afresh (e.g. NUM, NUM, NUM, GER, GER, GER, SG, SG, SG, NUM, NUM, NUM, GER; GER, GER, SG, SG, SG, NUM, …). Similar to the cue-based block, the rules had to be applied with an equally distributed probability (33.3%) but with a frequency of task switching of 33.3%. Sequences with the same cue in the last trial as n-1 trials before were categorized as repetition condition. Trials where this was not the case, were categorized as switch condition.

### 2.5 EEG recording and analysis

The EEG data were recorded using QuickAmp and BrainAmp amplifiers with 60 Ag/AgCl electrodes dispersed evenly over an elastic cap (ground electrode at coordinates θ = 58, ϕ = 78; reference electrode at coordinates θ = 90, ϕ = 90) at a 500 Hz sampling rate and later downsampled to 256 Hz. Electrode impedances were kept under 20 kΩ. EEG preprocessing was performed using automagic (Pedroni et al., 2018) and EEGLAB (Delorme and Makeig, 2004) in Matlab (2019a) (The MathWorks Corp.). Flat channels (near-constant signal) were first removed, and the EEG data were re-referenced to an average reference. The PREP preprocessing pipeline (Bigdely-Shamlo et al., 2015) and the EEGLAB “clean_rawdata()” pipeline were then applied. PREP removes line noise at 50 Hz using a multitaper algorithm and applies a robust average reference after removing contaminations by bad channels (excessive noise or artifacts). “clean_rawdata()” detects and removes flat-line, noisy, and outlier channels, reconstructs epochs showing abnormally strong power using Artifact Subspace Reconstruction (ASR (Mullen et al., 2013);), and removes time windows that cannot be reconstructed. A lowpass filter of 40 Hz (sinc FIR filter; order: 86 (Widmann et al., 2015);) was then applied. Electro-oculography artifacts were removed using a subtraction method (Parra et al., 2005). Residual muscle and eye artifacts were automatically classified and removed using an independent component analysis (ICA) based Multiple Artifact Rejection Algorithm (MARA (Winkler et al., 2014, 2011);). Components containing cardiac artifacts were identified using ICLabel (Pion-Tonachini et al., 2019) and subsequently removed. Finally, all channels that were removed automatically were interpolated using a spherical method. After these corrections, data were segmented for every condition separately into stimulus-locked epochs ranging from −2 s to +2 s relative to target onset.

### 2.6 RIDE

The RIDE method (manual http://cns.hkbu.edu.hk/RIDE.htm; Ouyang et al., 2015, 2011) was used to process the EEG data that had been corrected for baseline using the RIDE toolbox (15) in Matlab (2019a) (The MathWorks Corp.). RIDE assumes that ERPs consist of multiple components with different latencies, each reflecting distinct stages of cognitive processing. The method decomposes and reconstructs single-trial ERPs, allowing analysis of trial-to-trial variability. Its main advantage is that it overcomes the limitations of conventional stimulus-locked ERP averages, which can be blurred by latency variability across trials. Such blurring can distort ERP waveforms and attenuate effects of interest. By separating components with static and variable latencies based on their timing and variability, RIDE provides a more precise temporal mapping of cognitive processes. Current Source Densities (CSD) are not crucial because RIDE separates component clusters only by their latency variability rather than by their scalp distributions and waveforms (Wolff et al., 2017b). RIDE derives three clusters, namely the S-cluster, the R-cluster, and the C-cluster, associated with stimulus-driven processes, motor response, and decision-making, respectively. Time markers are set to the time points of the respective stimulus and response onsets in the EEG. The RIDE decomposition is conducted separately for each electrode channel using a time window function to extract the waveform of each component. The time window used for the S-cluster was -200-500 ms for the response marker, for the R-cluster, it was from -500-500 ms, and for the C-cluster, it was from 200 to 900 ms. Further details can be found in the.

### 2.7 Multivariate pattern analysis

The MVPA of the RIDE-decomposed data was conducted using the MVPA-light toolbox (Treder, 2020) in Matlab (2019a) (The MathWorks Corp.) as done by Takács et al. (2022b).

To determine when EEG activity could reliably distinguish between task repetition and task switching, group-level decoding was performed. Classification performance was measured as the area under the curve (AUC) across time points (diagonal of the decoding matrix). High AUC values indicate that neural patterns systematically differ between conditions at a given moment in time. This approach focuses on when differences between conditions emerge during the trial.

To assess the stability and generalizability of task-specific neural representations across time, temporal generalization analysis was conducted. Here, the classifier was trained at one time point and tested at all other time points, resulting in a time-by-time decoding matrix (training time × testing time). Accuracy along the diagonal reflects time-specific discriminability, similar to group-level decoding, whereas off-diagonal accuracy reflects the persistence or generalization of neural representations across time. This provides insights into the temporal dynamics and stability of neural patterns underlying cognitive flexibility over the entire trial.

To perform both analyses, we first decoded task repetition versus task switch trials separately for each of the three RIDE-decomposed clusters, in order to examine the neurophysiological representation of task switching. Next, temporal generalization MVPA was performed based on the repetition and switch class differences in the decomposed clusters to examine the temporal dynamics and the representational stability of task switching. The analyses were performed separately for the (i) mindfulness group before the intervention, (ii) mindfulness group after the intervention, (iii) audiobook group before the intervention, and (iv) audiobook group after the intervention in each temporally decomposed cluster. While binary decoding entailed performing training and testing on the same time points, temporal generalization also involved testing the time points of training on all other time points. The number of trials in the two classes was balanced with under-sampling to avoid overfitting (Treder, 2020). Classification features comprised EEG data at sixty channels in both stimulus classes. The decoding and temporal generalization were executed for each individual and each RIDE-cluster while maintaining the same parameters. To decode task repetition and task switch, an L1-Support Vector Machine (SVM) was employed as a classifier, as it performs better than the default linear discriminant analysis when the data is non-Gaussian, noisy, or prone to outliers (Treder, 2020). The SVM classifications were cross-validated with five folds, where data was split into five equal parts, and in each iteration step, one part was used for testing, and the remaining parts were used for training. The average of the iterations was calculated after each fold was used for testing. To identify the time interval showing the difference between the repetition and switch classes, the area under the curve (AUC) was used to measure decoding performance, and its performance was compared against the chance level of AUC = .5. Wilcoxon-tests were performed for each time point across participants to compare the decoding performance against the chance level. The cluster-based permutation method with 1000 permutations was used as a statistical correction method.

Taken together, group-level decoding identifies when neural activity patterns reliably differentiate switch versus repetition trials across participants whereas temporal generalization shows the stability and evolution of these task-specific representations The analyses were conducted separately for the mindfulness intervention group and the audiobook control group, before and after the intervention in the stimulus-related (S-cluster), intermediate (C-cluster), and response-related (R-cluster) cluster. This approach allows disentangling overlapping cognitive processes and provides a temporally precise view of the dynamics of cognitive flexibility during task switching. The main text presents the MVPA results based on data from N = 23 participants in each group. MVPA results of the whole sample (N = 30 in the mindfulness group and N = 23 in the audiobook group) are presented in Figure A3 and Figure A4.

### 2.8 Source localization analysis

In order to investigate the functional neuroanatomical sources linked to the sub-processes of switch costs during task switching, sLORETA (standardized low-resolution brain electromagnetic tomography) (Pascual-Marqui, 2002) using the original sLORETA software (Key Institute for Brain-Mind Research, University of Zurich) was used to analyze data exclusively from the Mindfulness group, which demonstrated significant temporary generalization performance in the MVPA process. The analysis was conducted using the standard sLORETA implementation without specifying an explicit SNR-based regularization parameter (Standardized LORETA = TRUE; Exact LORETA = FALSE; NoReg = TRUE; AutoReg = FALSE; SnrReg = FALSE; RelReg = FALSE). The sLORETA analysis aimed to identify differential modulations in the C-cluster between switch and repetition conditions, based on the time points selected in accordance with the temporary generalization results in the MVPA process (300-400ms). sLORETA is a reliable source localization method (Dippel and Beste, 2015; Ocklenburg et al., 2018; Sekihara et al., 2005) that partitions the intracerebral volume into 6239 voxels, resulting in a spatial resolution of 5 mm, and calculates standardized current density for each voxel. To conduct statistical comparisons, sLORETA images were generated for different contrasts using voxel-wise randomization tests based on statistical non-parametric mapping (SnPM) with 2000 permutations (https://www.uzh.ch/keyinst/NewLORETA/sLORETA/sLORETA.htm). Voxels with significant differences (p < .05, corrected for multiple comparisons) between contrasted conditions were located in the MNI-brain template (Colin27 T2). While EEG source reconstruction has limited spatial resolution and may require validation by functional imaging, it is the most suitable method for investigating the temporal process of neural activity across different brain structures in the current study due to its combination of time and spatial resolution.

### 2.9 Statistics

We used IBM SPSS Statistics (Version 28.0.1.1) to analyze symptom and behavioral data. The symptom data were analyzed by mixed effects repeated measure ANOVAS with the within-subject factor “Appointment” (pre vs. post) and between-subject factor “Group” (mindfulness vs. audiobook group). In regards to the task switching paradigm, we removed the first two trials of each block, all trials with an error, and the two trials following an error from both the behavioral and neurophysiological data. We eliminated trials with RTs greater than 2500 ms or less than 100 ms from the remaining trials. Behavioral data were analyzed using mixed effect ANOVA. This model included the within subject factor “Condition” (repeat vs. switch), “Block” (cue-based vs. memory-based), “Appointment” (pre-vs. post) and the between-subject factor “Group” (mindfulness vs. audiobook). Greenhouse–Geisser corrections were applied. All post-hoc tests were Bonferroni-corrected. Neurophysiological data were analyzed using MATLAB and sLORETA, as described in the respective paragraphs. After pre-processing the EEG data, 23 participants remained in the audiobook group for the MVPA analysis. To facilitate comparability between group-level decoding results, sample sizes were evened by removing 9 participants from the mindfulness group with the lowest trial numbers.

## 3 Results

### 3.1 Behavioral data

#### 3.1.1 Reaction times and switch costs

The mixed-effects ANOVA revealed main effects of (i) “Appointment” [*F*(1,51) = 10.827, *p* = .002, *η*^2^ = .175], indicating increased RTs during the pre-appointment (838.93 ms ± 25.77) versus post-appointment (772.12 ms ± 26.26), (ii) “Block” [*F*(1,51) = 25.974, *p* < .001, *η*^*2*^ = .337], indicating increased RTs during memory-based (830.65 ms ± 22.73) versus cue-based (780.39 ms ± 26.08) block and (iii) “Condition” [*F*(1,51) = 45.001, *p* < .001, *η*^*2*^ = .469], indicating increased RTs during the switch (832.77 ms ± 25.61) versus repetition condition (778.27 ms ± 222.91). The latter effects were further specified by a significant interaction of “Block x Condition” [*F*(1,51) = 21.344, *p* < .001, *η*^*2*^ = .295], indicating increased RTs during the switch versus repetition condition in both blocks, cue-based (794.14 ms ± 29.42 vs. 766.639 ms ± 28.75, *p* = .004) and memory-based (871.4 ms ± 24.72 vs. 789.9 ms ± 21.94, *p* < .001).

**Fig. 2:**
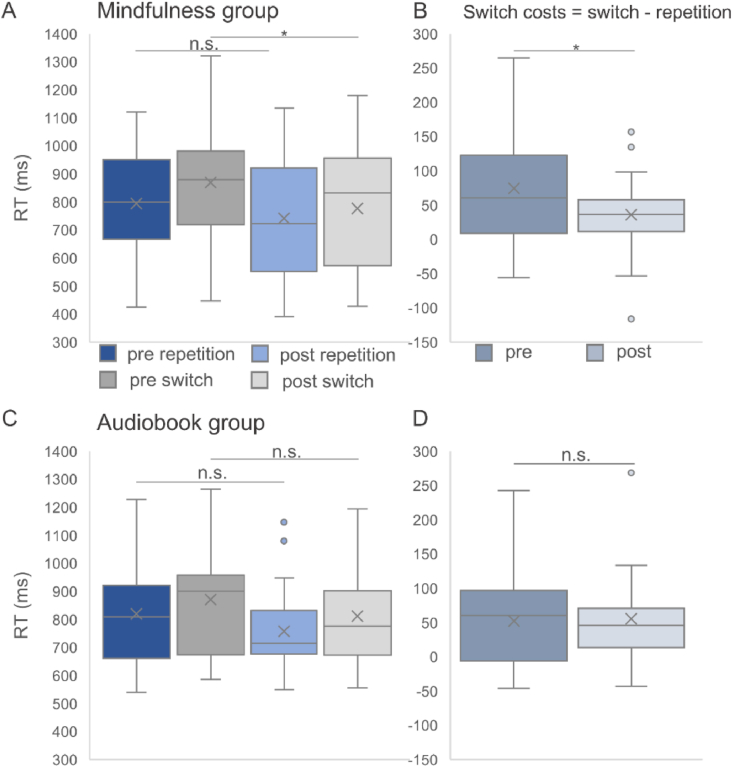
Behavioral data show (A) reaction times for the mindfulness group between the pre- and post-appointment for the different conditions (switch and repetition) averaged for the cue-based and memory-based block; (B) switch costs, comparing pre- and post-appointment in the mindfulness group; (C) reaction times for the audiobook group between the pre- and post-appointment for the different conditions (switch and repetition) averaged for the cue-based and memory-based block; (D) switch costs, comparing pre- and post-appointment in the audiobook group. Within each box, horizontal grey lines denote median values; boxes extend from the 25th to the 75th percentile of each condition's distribution of values; whiskers above and below the box indicate the 10th and 90th percentiles (i.e., the most extreme values within 1.5 interquartile range of the 25th and 75th percentile of each condition); dots denote observations outside the range of adjacent values.

We observed a significant overall interaction of “Appointment x Condition x Group” [*F*(1,51) = 5.481, *p* = .023, *η*^*2*^ = .097]. This interaction is shown in Fig. 2. In order to understand this interaction, repeated measures ANOVAs were conducted for the mindfulness and audiobook group separately. Only the mindfulness group [*F*(1,29) = 9.589, *p* = .004, *η*^*2*^ = .248], but not the audiobook group [*F*(1,22) = .073, *p* = .79, *η*^*2*^ = .003] showed a significant effect of “Appointment” x “Condition”. Post-hoc tests revealed that within the mindfulness group, there were decreased RTs during the post-appointment compared to the pre-appointment in the switch condition (777.3 ms ± 38.35 vs. 869.36 ± 39.97, *p* = .004), but not in the repetition condition (741.55 ms ± 38.33 vs. 794.74 ms ± 32.85, *p* = .078).

Concerning the switch costs (switch-repetition), there was a significant interaction of “Appointment x Group” [*F*(1,51) = 5.481, *p* = .023, *η*^*2*^ = .097]. A post-hoc test of the interaction revealed a significant effect of “Group”, showing significantly lower switch costs in the mindfulness group during the post- (35.74 ms ± 10.56) compared to the pre-appointment (74.62 ms ± 13.72, *p* = .002). In the audiobook group, no effect was observed (pre: 52.13 ms ± 15.67, post: 55.51 ms ± 12.06, *p* = .805). To examine whether comorbid diagnoses or medication status influenced behavioral outcomes, we included these factors as covariates in the mixed-effects ANOVA on switch costs. The analysis revealed no significant effect of comorbidity [F(1,481) = .219, p = .642, η^2^ = .004] or medication status [*F*(1,28) = .013, *p* = .909, *η*^*2*^ < .001]. Importantly, the “Appointment x Group” interaction remained significant [*F*(1,11051) = 5.031, *p* = .029, *η*^*2*^ = .093], indicating that the observed behavioral effects were robust and not confounded by comorbid diagnoses or medication.

#### 3.1.2 Hit accuracy

A mixed-effects ANOVA on accuracy (percentages of hits) yielded significant main effects of (i) “Appointment” [*F*(1,51) = 17.318, *p* < .001, *η*^*2*^ = .253], indicating higher accuracy during the post appointment (89.63 ± .85%) than during the pre-appointment (86.84 ± 1.08%), (ii) “Block” [*F*(1,51) = 5.898, *p* = .019, *η*^*2*^ = .104], indicating higher accuracy in the cue-based (89.1 % ± .8) than the memory-based block (87.37 % ± 1.13), and (iii) “Condition” [*F*(1,51) = 42.996, *p* < .001, *η*^*2*^ = .457], indicating higher accuracy during repetition (89.66 % ± .87) than during switch trials (86.81 % ± 1). There was a between-subject effect of “Group” [*F*(1,51) = 4.363, *p* = .042, *η*^*2*^ = .079], indicating a higher accuracy in the mindfulness group (90.13 % ± 1.2) compared to the audiobook group (86.34 % ± 1.37). No other effect or interaction was observed to be significant (all *p* > .078).

### 3.2 Neurophysiological data

#### 3.2.1 Multivariate results: group-level decoding and source localization

**Fig. 3:**
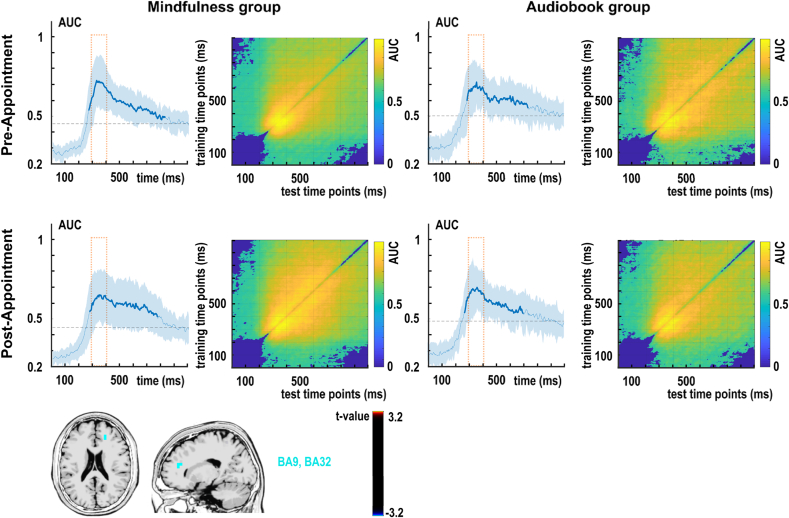
RIDE decomposed C-cluster EEG data for the classification of switch versus repetition condition (switch costs) for the mindfulness and audiobook group for the pre- and post-appointment. Left: Area under the curve (AUC) decoding accuracy across time. Time zero denotes the presentation of the target stimulus. Thicker blue lines indicate significant time windows (p < .05; two-sided cluster-based permutation). Right: Temporal generalization plot. The plots show the degree to which the classifier when trained on a given time point (y-axis) generalizes to time points in the trial (x-axis). The colors indicate the classifier performance. The diagonal (bottom left to top right) shows classification performance when the classifier is trained and tested simultaneously. Bottom: results of sLORETA source localization for significant time window 300-400ms in the mindfulness group between the pre- and post-appointment. Activity differences (against zero) were found in the medial frontal gyrus (BA9) and anterior cingulate (BA32).

Fig. 3 presents the decoding performance between task repetition and task switching conditions for the C-cluster data reflecting response selection mechanisms (Ouyang et al., 2011, 2015). In the mindfulness group's pre-appointment (before the intervention), classification was significantly above chance between 266 ms and 898 ms after stimulus onset. In the mindfulness group's post-appointment (after the intervention), classification was significantly above chance between 273 ms and 770 ms after the stimulus presentation. After inspecting the mindfulness group's C-cluster decoding results, a decrease in classification performance was observed between the two appointments in the interval of 300-400 ms (Fig. 3). This difference was confirmed by a paired samples *t*-test comparing individual AUC values in this time interval between appointments: t(22) = 1.743, p = .048, d = .364, one-tailed. In this time frame, the mindfulness group showed differential activation between the pre-and post-appointment regarding the switch costs in the medial frontal gyrus (BA9) and anterior cingulate (BA32) (Fig. 3). In the audiobook group's pre-appointment (before the intervention), classification was significantly above chance between 289 ms and 738 ms after stimulus onset. In the mindfulness group's post-appointment (after the intervention), classification was significantly above chance between 270 ms and 703 ms after the stimulus presentation.

**Fig. 4:**
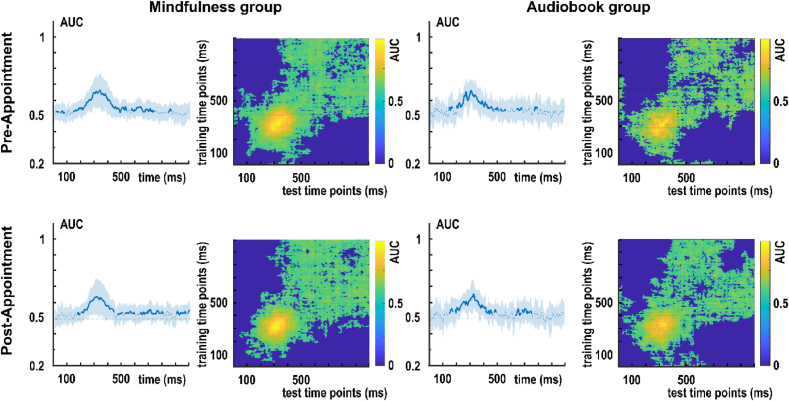
RIDE decomposed S-cluster EEG data for the classification of switch versus repetition condition (switch costs) for the mindfulness and audiobook group for the pre- and post-appointment. Left: Area under the curve (AUC) decoding accuracy across time. Time zero denotes the presentation of the target stimulus. Thicker blue lines indicate significant time windows (p < .05; two-sided cluster-based permutation). Right: Temporal generalization plot. The plots show the degree to which the classifier when trained on a given time point (y-axis) generalizes to time points in the trial (x-axis). The colors indicate the classifier performance. The diagonal (bottom left to top right) shows classification performance when the classifier is trained and tested simultaneously.

Fig. 4 presents the decoding performance between task repetition and task switching conditions for the S-cluster data. In the mindfulness group's pre-appointment, classification was significantly above chance in the time intervals of 12-35 ms, 47-78 ms, 141-531 ms, 566-734 ms, and 840-859 ms relative to stimulus onset. In the mindfulness group's post-appointment, classification was significantly above chance in the time intervals of 172-191 ms, 199-441 ms, 465-504 ms, 520-586 ms, 633-805 ms, 902-973 ms, 984-1000 ms relative to stimulus onset. In the audiobook group's pre-appointment, classification was significantly above chance in the time intervals of 152-180 ms, 207-481 ms, 488-516 ms, 523-543 ms, 551-570 ms, and 648-691 ms relative to stimulus presentation. In the audiobook group's post-appointment, classification was significantly above chance in 156-184 ms, 195-467 ms, 481-500 ms, 641-680 ms, and 688-762 ms after the stimulus presentation.

**Fig. 5:**
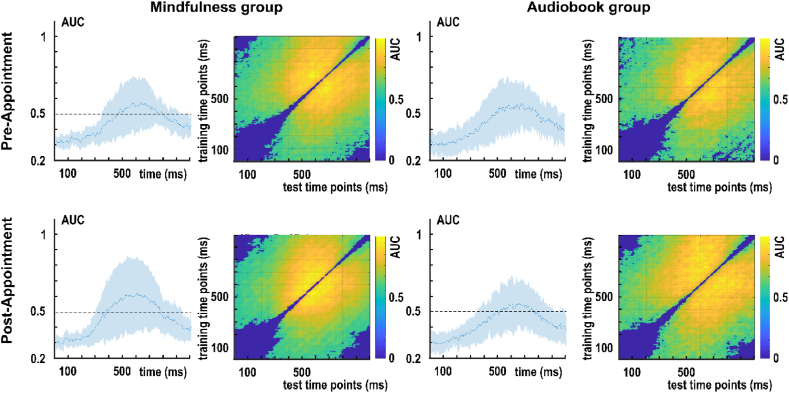
RIDE decomposed R-cluster EEG data for the classification of switch versus repetition condition (switch costs) for the mindfulness and audiobook group for the pre- and post-appointment. Left: Area under the curve (AUC) decoding accuracy across time. Time zero denotes the presentation of the target stimulus. No significant time windows (p < .05; two-sided cluster-based permutation) were found. Right: Temporal generalization plot. The plots show the degree to which the classifier when trained on a given time point (y-axis) generalizes to time points in the trial (x-axis). The colors indicate the classifier performance. The diagonal (bottom left to top right) shows classification performance when the classifier is trained and tested simultaneously.

In the R-cluster, decoding was unsuccessful, that is, classification results did not differ significantly from the chance level in either group and appointment (Fig. 5).

MVPA analyses provided successful classifications for the C-cluster and S-cluster but not for the R-cluster data. Thus, neurophysiological representations of task switching could be observed at the stimulus-related and stimulus-response translational coding levels. The latter revealed a decrease in decoding performance in the time window of 300-400 ms, which was specific to the mindfulness group. Topographical scalp maps for the time windows showing significant effects above chance classification after stimulus onset can be found in Appendices 4.

#### 3.2.2 Multivariate results: temporal generalization

Fig. 3 also presents the temporal generalization results for the C-cluster data. In both groups and appointments, decoding accuracy was highest (i.e., AUC >.65) along the diagonal of the matrices between 200 ms and 300 ms after stimulus presentation. That is, the neurophysiological signal of task switch and task repetition classes were highly generalizable to each other within the 200-300 ms time window. Decoding performance gradually decreased towards the axes of the matrices. However, it remained significantly above the chance level, except for the edges in the bottom left, top left, and bottom right corners (Fig. 3). Notably, between ∼400 and 800 ms, the AUC over the diagonal decreased, then after ∼800 ms, diagonal decoding was non-significant in all four matrices. That is, the late stage of the trial window did not generalize between task switch and task repetition classes. However, off-diagonal decoding remained significant in all four analyses.

Fig. 4 depicts the temporal generalization matrices for the S-cluster data. In both groups and appointments, decoding accuracy was highest (i.e., AUC >.6) over the diagonal between 200 ms and 480 ms after stimulus onset. In all four matrices, the central decodings were temporally jittered both in the onset and offset. Additionally, off-diagonal ramping was observed after ∼600 ms in both groups and appointments. Notably, this ramping pattern showed larger asymmetry between the off-diagonal parts in the audiobook group's post-appointment than in the mindfulness group's post-appointment.

Fig. 5 presents the temporal generalization results for the R-cluster data. In the R-cluster, significant classification occurred only in off-diagonal but not in diagonal parts of the matrices (c.f. *Group-level decoding results*). Decoding accuracy was highest (AUC >.6) in the time window of ∼600-800 ms, which corresponds to the average response times in the task (c.f., Behavioral results 3.1.1).

In sum, C-cluster generalization matrices were characterized by a single decoding: it was strongest along the diagonal between 300 and 400 ms and was smoothed in both horizontal and vertical directions by a temporal jitter in the offset. MVPA-based classification between task switching and task repetition classes showed distinctive patterns of temporal generalizability in the three coding levels. C-cluster and S-cluster results suggested a singular representational activity that peaked between 300 and 400 ms. However, S-cluster classifications were less accurate than in the C-cluster, and the off-diagonal activities were also shorter. That is, task switching was observed as a more transient representation at the stimulus coding level than at the stimulus-response translational level. At the response coding level, the peak occurred in the time window of response execution. Unlike in the C-cluster and S-cluster data, R-cluster temporal generalization matrices only showed an off-diagonal distinction between task switch and task repetition.

### 3.3 Symptom data: CY-BOCS

The repeated measures ANOVA revealed a significant effect on the CY-BOCS score for “Appointment” (pre vs. post) [*F*(1,51) = 24.482; *p* < .001; *η*^*2*^ = .324], showing that the score was higher at the pre-appointment (16.25 ± .89) than at the post-appointment (12.54 ± .84). There was no interaction effect with “Group”; *F*(1,51) = .107; *p* = .745; *η*^*2*^ = .002. Comparisons of the mean between the pre- and post-appointment in the mindfulness and audiobook groups can be found in Table A1.

## 4 Discussion

This study investigated the effects of an app-based mindfulness meditation training on cognitive flexibility in adolescents with OCD, using behavioral measures (i.e., switch costs) and neurophysiological data from MVPA of RIDE-decomposed EEG signals. To our knowledge, this is the first study to examine the impact of a mindfulness-based intervention on cognitive flexibility in an adolescent sample with OCD using a temporally sensitive neurophysiological approach.

Behaviorally, adolescents in the mindfulness group exhibited a significant reduction in switch costs following the 8-week intervention, primarily due to faster reaction times in the switch condition. This improvement was not observed in the active control group, suggesting that mindfulness training may help to better focus on the present moment and inhibit engaging in further processing of irrelevant content (previous task). This way, one's attention is on the current task, thus performing faster in a more difficult task (as in the switch condition compared to the repetition condition). These findings align with prior research indicating that mindfulness training is related to improved attentional functions and cognitive flexibility (Chambers et al., 2008; Moore and Malinowski, 2009; Vieth and von Stockhausen, 2022). Our findings also build upon a previous study using the same task-switching paradigm. Differences in switch costs have been reported between healthy adolescents and those with OCD (Wolff et al., 2017a), between healthy children and young adults (Wolff et al., 2016), and in adults following a brief mindfulness meditation (Wolff and Beste, 2020). Interestingly, in the current study, participants did not exhibit differential switch costs between cue-based and memory-based blocks after the intervention, suggesting that mindfulness training reduced switch costs equally across both cognitive flexibility demands. Importantly, the observed behavioral effects were not confounded by medication or comorbidity.

Neurophysiologically, both groups demonstrated significant above-chance classification of switch versus repetition trials in both the pre- and post-appointment periods in the C- and S-clusters, but not in the R-cluster, suggesting that task-switching representations in OCD adolescents are most prominently encoded at stimulus-related and stimulus-response integration levels. Importantly, we observed a decrease in decoding performance between 300 and 400 ms post-stimulus following the intervention in the mindfulness group only in the C-cluster, which was not present in the control group. This decrease should not be misinterpreted as a loss of function. Instead, it likely reflects a desired outcome: a normalization of neural processing, such that the neural patterns associated with switch and repetition trials become more similar. This supports the idea that the intervention reduced the atypical cognitive processing seen in OCD. Task-switching processes have previously been associated with the C-cluster (V. Petruo et al., 2019; V.A. Petruo et al., 2019; Wolff et al., 2017a; Wolff and Beste, 2020), which mediates the transition between stimulus evaluation and response execution (Verleger et al., 2014). Smaller reaction time differences (smaller switch costs) after the intervention indicate reduced cognitive load. This could be associated with more similar neural patterns underlying switch and repetition trials. However, exploratory analyses examining the association between changes in behavioral switch costs and neurophysiological differences did not reveal a significant relationship (r(21) = −.24, p = .26). This lack of a significant correlation may be partly due to the limited sample size and resulting lower statistical power. Thus, while the findings are consistent with the notion that mindfulness meditation may influence neural mechanisms responsible for updating internal task sets during response selection, the relationship between behavioral and neurophysiological changes remains tentative.

Source localization identified this normalization in neural discrimination as occurring in the medial frontal gyrus (BA9) and anterior cingulate cortex (BA32). The medial frontal gyrus has been reported to be active in an fMRI study of switching (Rushworth et al., 2002) and during context switching compared to perceptual and response switching (Kim et al., 2012). Notably, the ACC has been consistently implicated in cognitive control (Brockett and Roesch, 2021; Devinsky et al., 1995; Huster et al., 2007; Shenhav et al., 2024; Vanveen and Carter, 2002), and in the pathophysiology of OCD (Liu et al., 2022; Shim et al., 2009; Ursu et al., 2003). In individuals with OCD, the ACC frequently shows both functional and structural abnormalities, including hyperactivity during error processing (Ursu et al., 2003), reduced cortical folding (Shim et al., 2009), and altered resting-state connectivity (Liu et al., 2022). This dysregulation contributes to the inflexible, repetitive behaviors and cognitive rigidity that characterize the disorder. In this context, the observed modulation of ACC activity following mindfulness training in our study is significant. In line with previous findings, brief focused attention meditation in healthy adults has also been shown to modulate ACC activity during task switching using the same paradigm (Wolff and Beste, 2020). Additionally, a meta-analyses and recent reviews indicate that the ACC is among the areas most consistently altered in mindfulness meditation practitioners, supporting their central role in the neural response to mindfulness training (Fox et al., 2014; Tang et al., 2015; Zsadanyi et al., 2021). The observed activation changes in this study suggest that these regions may serve as neural hubs integrating cognitive control and mindfulness-related regulatory processes, particularly in the context of switching between mental sets, and indicate a normalization of these previously dysregulated mechanisms. Further, the mindfulness training may have led to more efficient or less effortful engagement of these brain regions during task switching. This is consistent with theories positing that mindfulness enhances present-moment awareness and reduces cognitive rigidity (engaging in further processing of irrelevant content, which is a hallmark of OCD) by promoting adaptive regulation of attention and internal state representations (Gu et al., 2015; Kabat-Zinn, 2003; Segal et al., 2018).

Although both groups showed significant reductions in CY-BOCS scores over time, there was no interaction between group and appointment. This implies that while both interventions may have had a general therapeutic benefit, only mindfulness training had a specific impact on cognitive flexibility as measured behaviorally and neurophysiologically. The lack of group differences in symptom reduction may also reflect shared component during both interventions such as structured daily engagement or increased self-monitoring, while effects of ongoing psychotherapy in some participants, which remained constant during the intervention period, could also have contributed to symptom changes.

A subset of participants was excluded due to challenges inherent to longitudinal EEG studies in children and adolescents. Nevertheless, the retained sample offers high-quality, longitudinal neurophysiological data, which are rare in this population and provide valuable insights into the effects of mindfulness interventions.

In conclusion, this study provides converging behavioral and neurophysiological evidence that a mindfulness meditation training can enhance cognitive flexibility in adolescents with OCD. These effects were particularly evident in the stimulus-response integration stage of task switching and were associated with altered activation in brain regions involved in cognitive flexibility. In contrast, no significant decoding or behavioral changes were observed in the audiobook control group. This suggests that simple engagement with structured auditory content, although potentially relaxing or enjoyable, was insufficient to affect cognitive flexibility or its neural correlates in the way mindfulness training did. The use of an active control group strengthens our findings by reducing the likelihood that the observed effects were due to non-specific intervention factors. The findings highlight that mindfulness-based interventions selectively modulate task-switching processes, as evidenced by MVPA of ACC activity, suggesting potential avenues for adjunctive interventions in OCD that warrant further investigation.

## CRediT authorship contribution statement

**Sarah Rempel:** Writing – original draft, Visualization, Validation, Software, Project administration, Methodology, Investigation, Formal analysis, Data curation. **Maria McDonald:** Writing – review & editing, Investigation. **Veit Roessner:** Writing – review & editing, Resources. **Christian Beste:** Writing – review & editing, Supervision, Resources, Methodology, Conceptualization. **Nicole Beyer:** Writing – review & editing, Validation, Supervision, Software, Methodology, Funding acquisition, Conceptualization.

## Declaration of competing interest

Prof. Veit Roessner has received lecture honoraria from Infectopharm and Medice companies. He has carried out clinical trials in cooperation with Servier and Shire Pharmaceuticals/Takeda companies and has received financial research support or compensation from the government entities of the DFG and BMBF. The present work is unrelated to the above grants and relationships. All other authors declare no conflict of interest.

## Data Availability

Data presented in the study can be found under the following link: https://osf.io/8vfnj/
