# Supplementary material for: App-based mindfulness meditation training enhances cognitive flexibility and modulates ACC and medial frontal gyrus activation during task switching in adolescent OCD
Source: Neuroimage Rep. 2026 Apr 23;6(2):100347. doi: 10.1016/j.ynirp.2026.100347 (PMC13127193; doi:10.1016/j.ynirp.2026.100347)
Supplement: Multimedia component 1 [file mmc1.docx]

**Appendices 1**

Table A1 shows exclusion details of the included participants (N = 76).

Table A1: Exclusion details of all included participants (N = 76).

| **Reason for exclusion** | **Number of excluded participants in the Mindfulness group (N = 44)** | **Number of excluded participants in the Audiobook group (N = 32)** |
| --- | --- | --- |
|  |  |  |
| Problem during EEG recording | 3 (Participant_004, _104, _071 too many mistakes, paradigm stopped) | 2 (participant _105 went to the bathroom, _068 EEG gel was itching) |
| Drop out | 6 (Participants _006, _007, _017, _083, _090, _103) | 0 |
| Exclusion after pre-processing EEG-data with Automagic | 1 (Participant _086) | 4 (Participants _052, _048, _084, _056) |
| Low compliance using app | 2 (Participants _093, _098) | 0 |
| No post appointment due to Corona | 0 | 1 (Participant _040) |
| Extreme outlier Behavior (> ± 3*interquartile range) | 1 (Participant _097 in POST_Hit_PERCENT_cue_  rep_all_rules) | 1 (Participant _062 in POST_Hit_PERCENT_cue_  rep_all_rules) |
| Outlier (> ± 2SD) Switch Costs Difference Pre-Post | 1 (Participant _029) | 1 (Participant _037) |
| Total number (%) | 14 (31.82 %) | 9 (28.13 %) |

**Appendices 2**

Table A2 shows details about the final sample (N = 53).

Table A2: Details about the final sample (N = 53).

| **Final sample** | **Mindfulness group (N = 30)** | **Audiobook group (N = 23)** |  |
| --- | --- | --- | --- |
| OCD-Diagnosis |  |  |  |
| F42.0 | 1 (3.33 %) | 0 |  |
| F42.1 | 3 (10 %) | 2 (8.7 %) |  |
| F42.2 | 26 (86.66 %) | 19 (82.61 %) |  |
| F42.2 suspected | 0 | 2 (8.7 %) |  |
| Medication |  |  |  |
| Aripiprazole | 1 | 0 |  |
| Agomelatin | 1 | 0 |  |
| Escitalopram | 1 | 0 |  |
| Fluoxetine | 7 | 2 |  |
| Fluvoxamine | 0 | 1 |  |
| L-Thyroxin | 1 | 0 |  |
| Medikinet | 0 | 1 |  |
| Methylphenidate | 0 | 1 |  |
| Risperidone | 1 | 0 |  |
| Sertraline | 1 | 3 |  |
| Total number of participants taking medication (%) | 11 (36.67 %) | 6 (26.09 %) | *X^2^*(1) = .669, *p* = .413 |
| Comorbidities |  |  |  |
| F32.1 | 3 | 1 |  |
| F32.2 | 0 | 1 |  |
| F33.1 | 1 | 0 |  |
| F40.0 | 1 | 1 |  |
| F40.1 | 2 | 0 |  |
| F40.2 | 0 | 1 |  |
| F41.0 | 1 | 0 |  |
| F43.2 | 1 | 0 |  |
| F50.1 | 3 | 0 |  |
| F60.31 | 1 | 0 |  |
| F63.3 | 1 | 0 |  |
| F64.0 | 1 | 0 |  |
| F64.9 | 1 | 0 |  |
| F81.2 | 1 | 0 |  |
| F90.0 | 0 | 3 |  |
| F95.1 | 1 | 1 |  |
| F95.2 | 2 | 2 |  |
| F98.8 | 1 | 0 |  |
| Total number of participants with comorbidities (%) | 11 (36.67%) | 7 (30.43%) |  |
| Gender |  |  |  |
| number of female participants (%) | 18 (60 %) | 11 (47.83%) | *X^2^*(1) = .779, *p* = .378 |
| IQ |  |  |  |
| *M* (± *SD*) | 105.7 (± 14.42) | 98.24 (± 12.43) | *T*(51) = 1.98, *p* = .053 |
| Age |  |  |  |
| *M* (± *SD*) | 15.62 (± 2.2) | 15.12 (± 1.93) | *T*(51) = .855, *p* = .397 |
| CY-BOCS |  |  |  |
| PRE *M* (± *SD*) | 18.23 (± 7.11) | 14.26 (± 5.38) | *T*(51) = 2.233, *p* = .03 |
| POST *M* (± *SD*) | 14.77 (± 6.59) | 10.3 (± 6.09) | *T*(51) = 2.523, *p* = .015 |
| App usage |  |  |  |
| Total in min (± *SD*) | 776.93 (± 303.88) | 1121.39 (± 278.69) | *T*(51) = -4.238, *p* < .001 |

Appendices 3

The main text presents the MVPA results based on data from N = 23 participants in each group. MVPA results of the whole sample (N = 30 in the mindfulness group and N = 23 in the audiobook group) are presented. Figure A3.1 shows Group-level decoding results, Figure A3.2 Temporal generalization matrices.

*
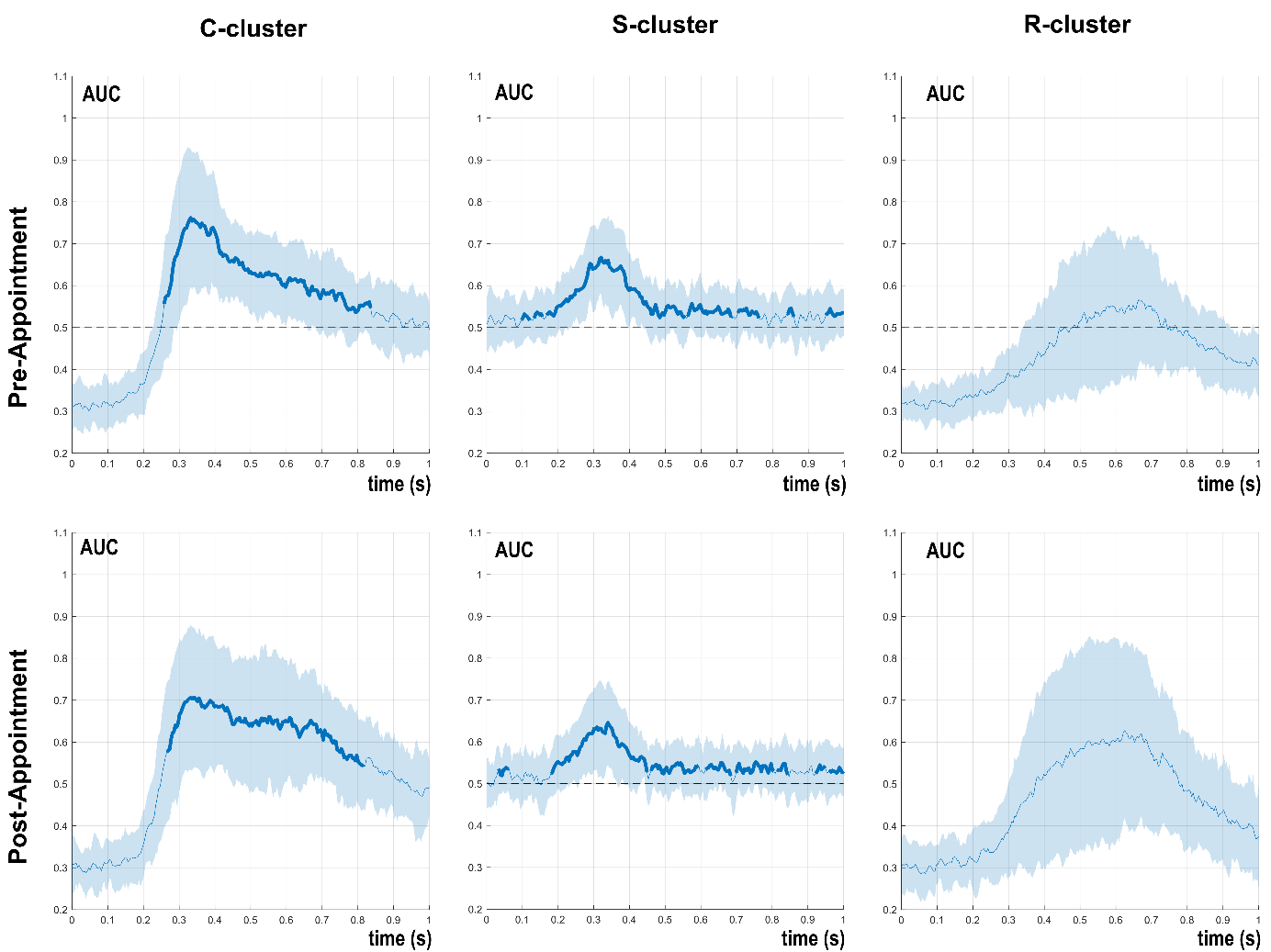
*

**Figure A3:** Group-level decoding results in the complete (N = 30) mindfulness group. The left side presents the decoding performance between task repetition and task switching conditions for the C-cluster data. The middle column presents the decoding performance between task repetition and task switching conditions for the S-cluster data. The right side presents decoding results for the R-cluster data. Time zero denotes stimulus presentation. Thick lines represent classification accuracy significantly above chance level.


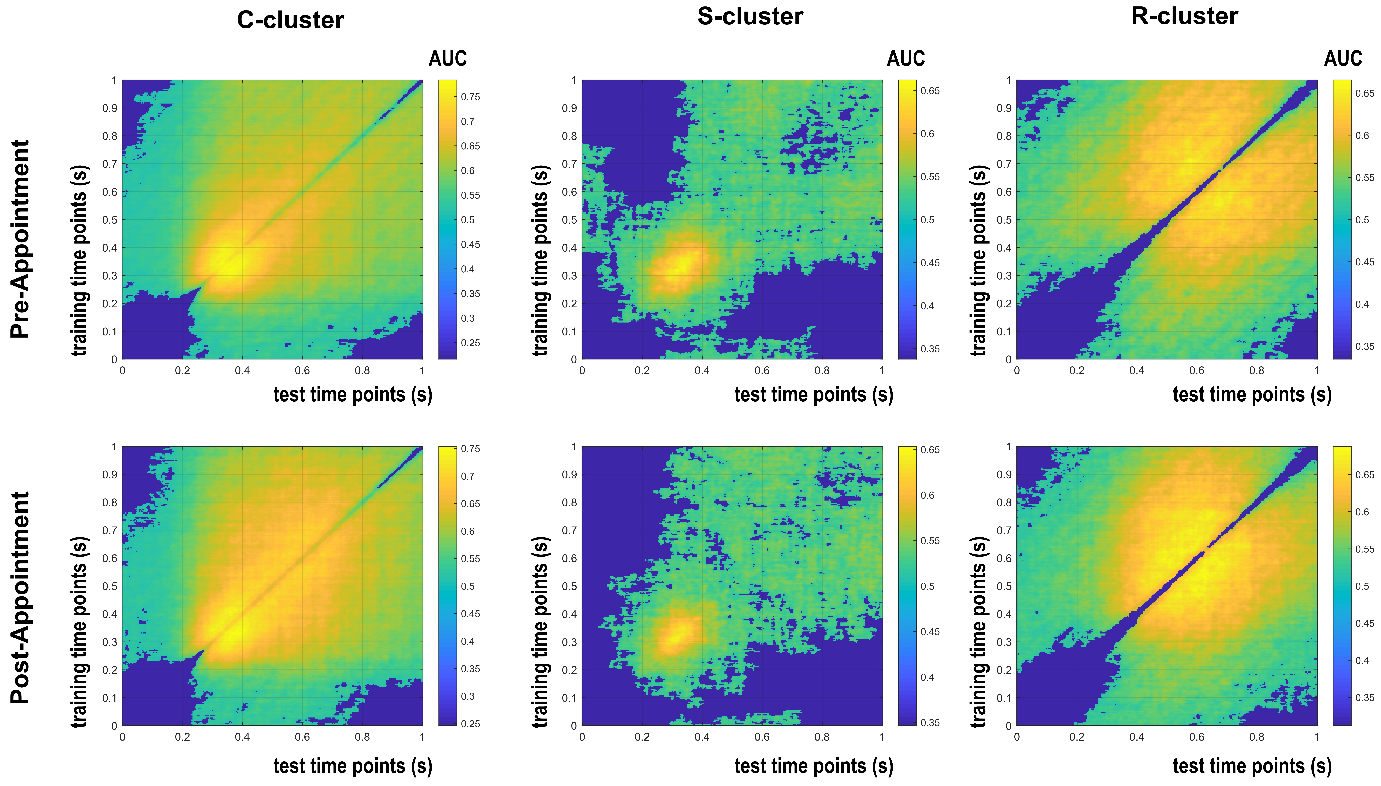


**Figure A4:** Temporal generalization matrices in the complete (N = 30) mindfulness group. The left side presents the temporal generalization results for the C-cluster data. S-cluster results are depicted in the middle. The right side presents temporal generalization for the R-cluster data. Time zero denotes stimulus presentation.

Appendices 4

Topographical scalp maps for the time windows showing significant effects above chance classification after stimulus onset.

**C-cluster**

Mindfulness group pre-appointment

| 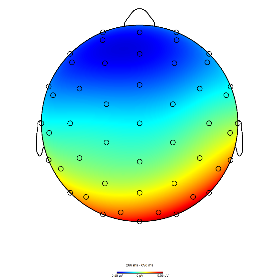 | 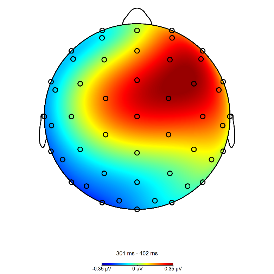 |
| --- | --- |
| 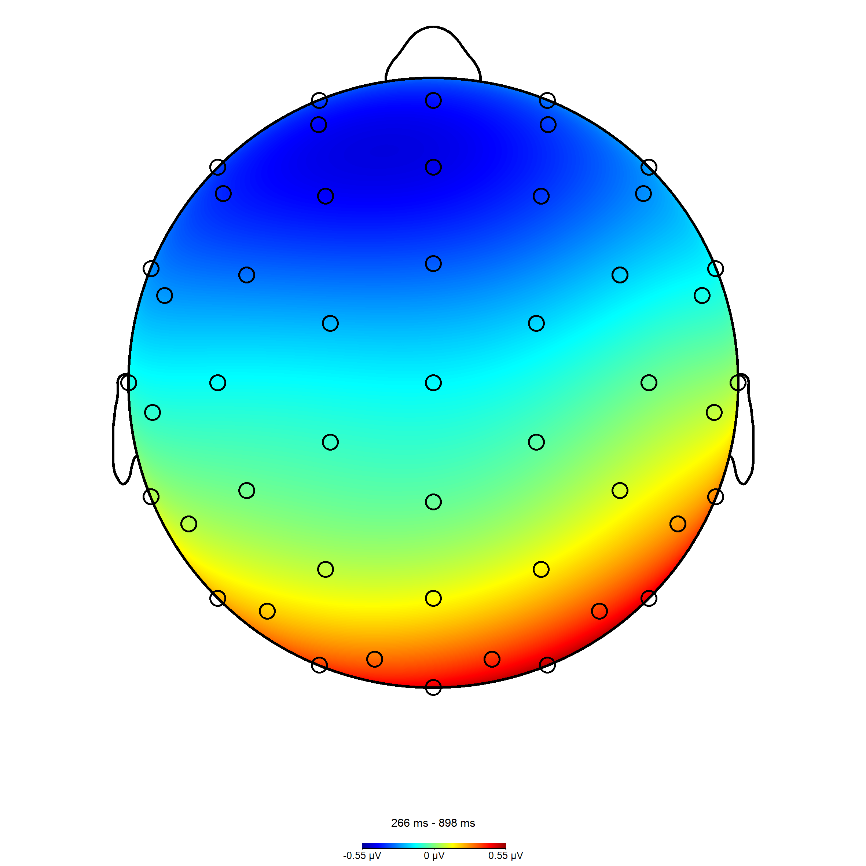 | 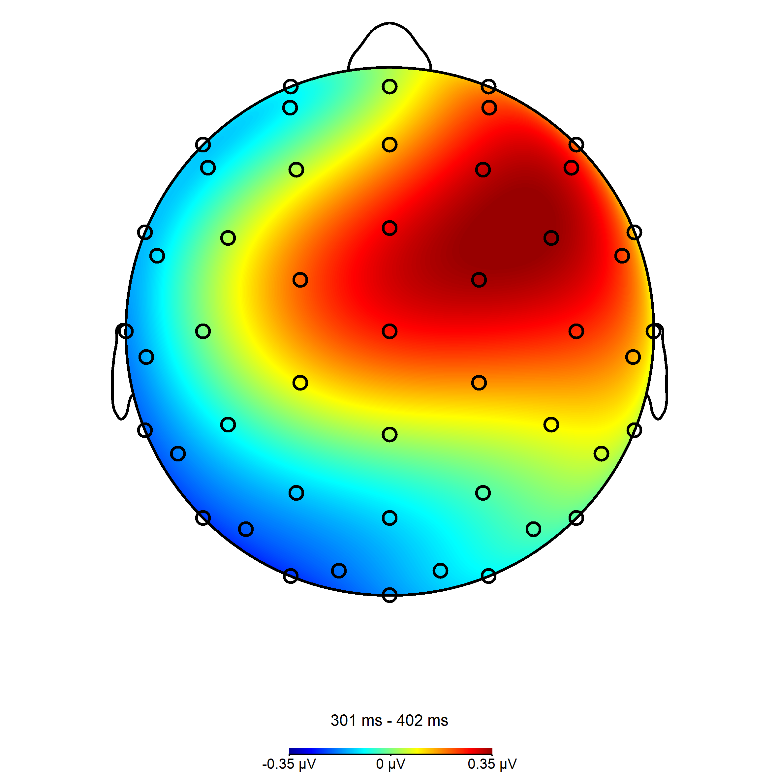 |

Mindfulness group post-appointment

| 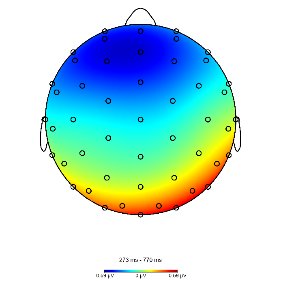 | 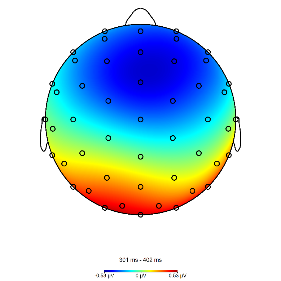 |
| --- | --- |
| 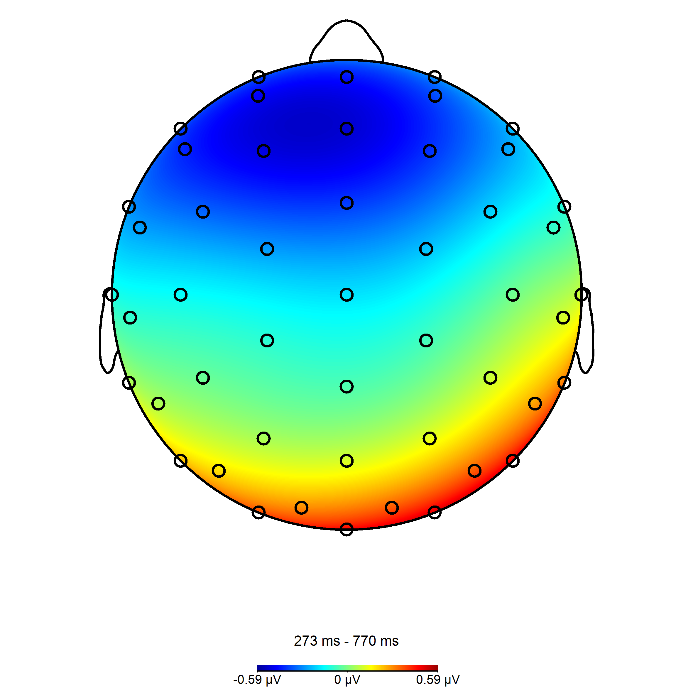 | 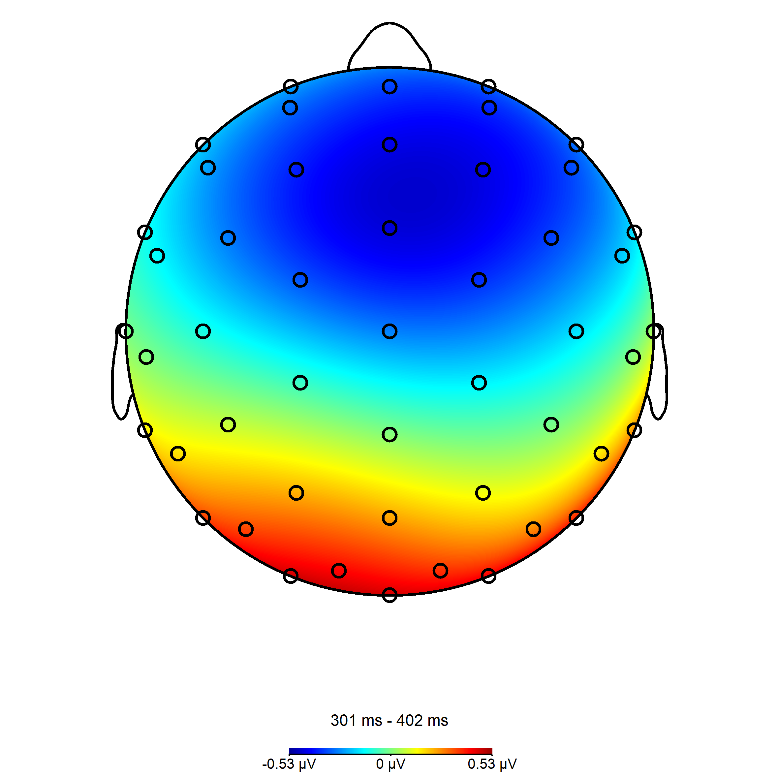 |

Audiobook group pre-appointment

| 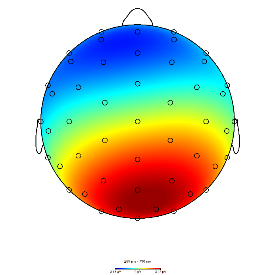 |  |
| --- | --- |
| 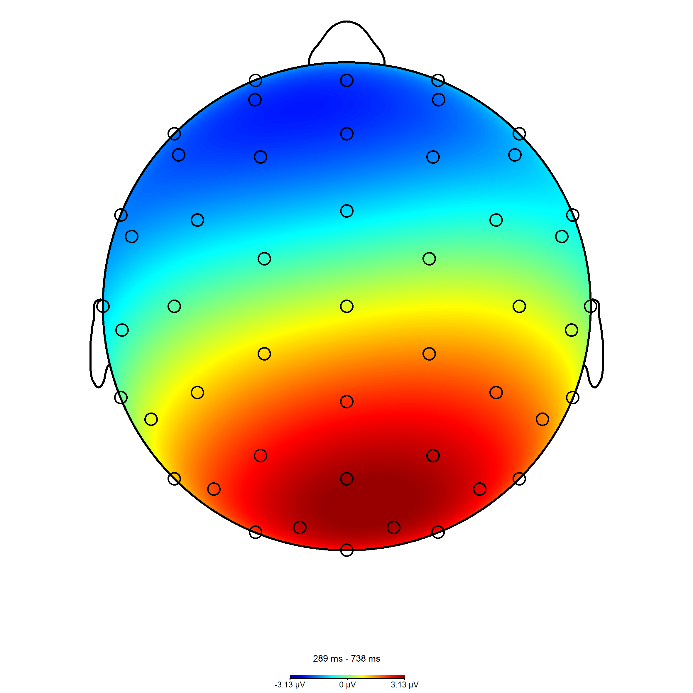 |  |

Audiobook group post-appointment

| 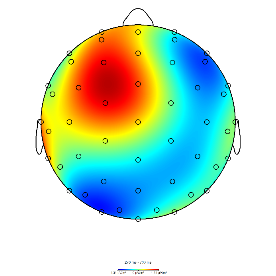 |
| --- |
| 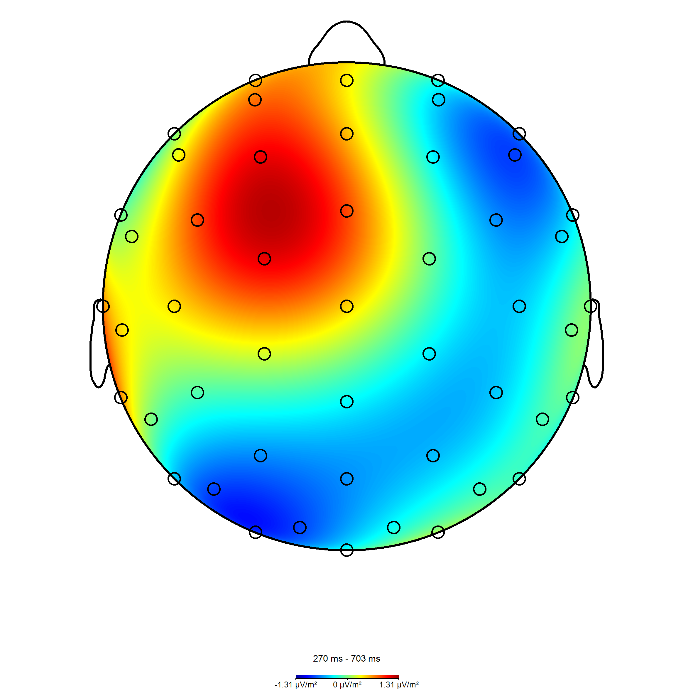 |

**S-cluster**

Mindfulness group’s pre-appointment

| 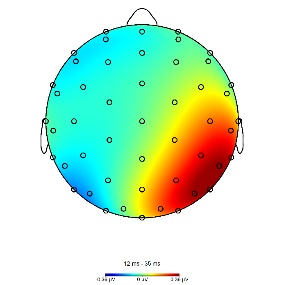 | 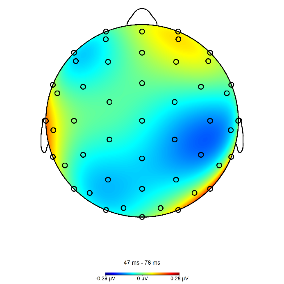 | 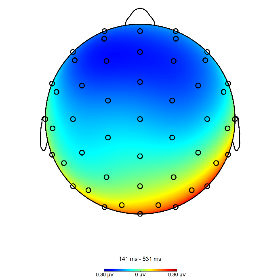 | 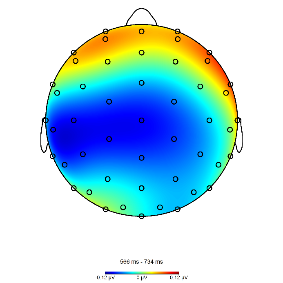 | 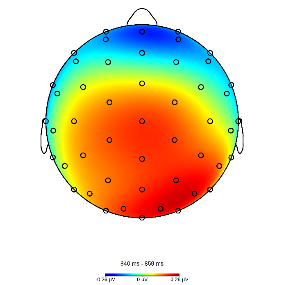 |
| --- | --- | --- | --- | --- |
| 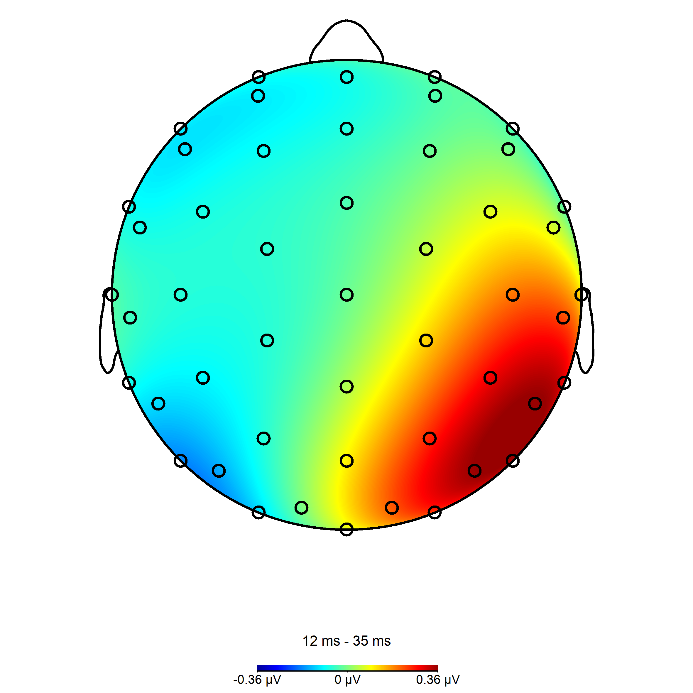 | 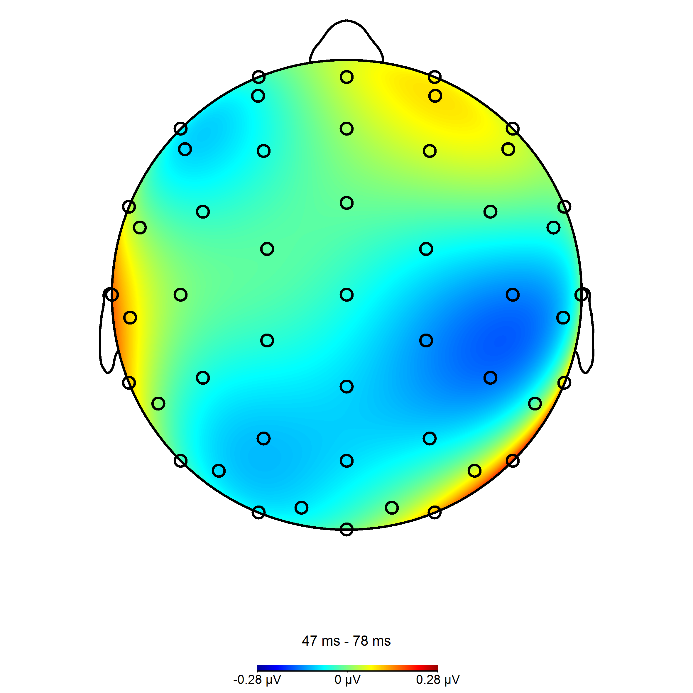 | 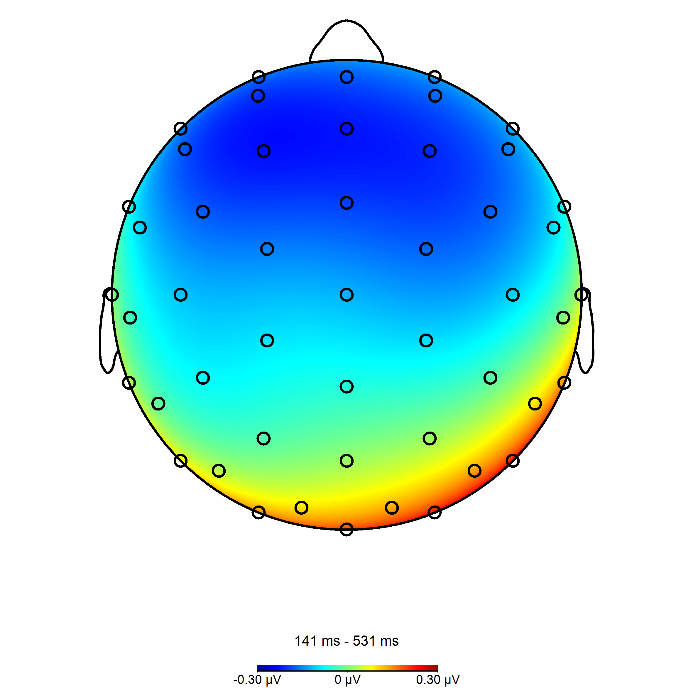 | 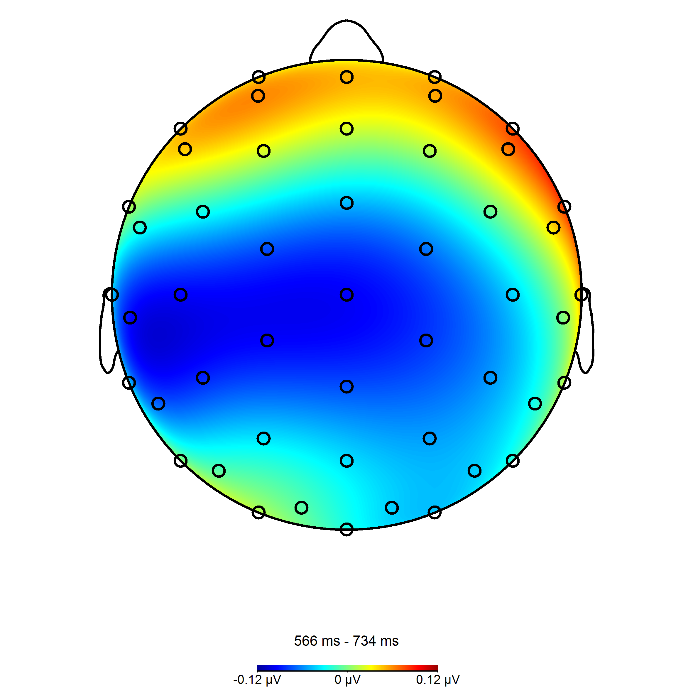 | 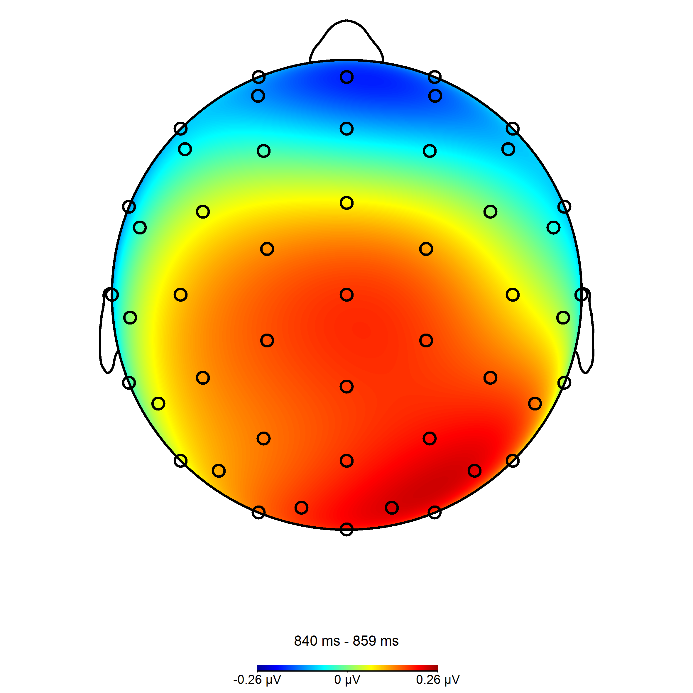 |

Mindfulness group post-appointment

| 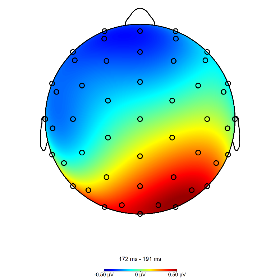 | 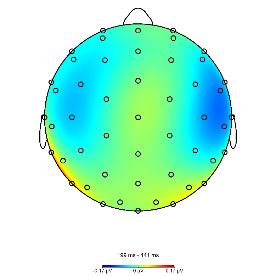 | 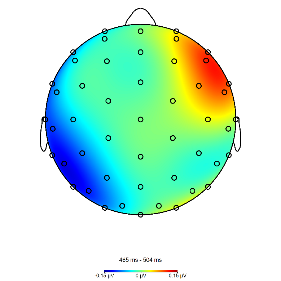 | 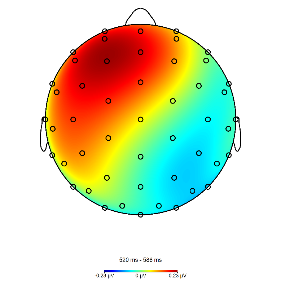 | 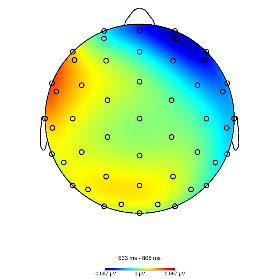 |
| --- | --- | --- | --- | --- |
| 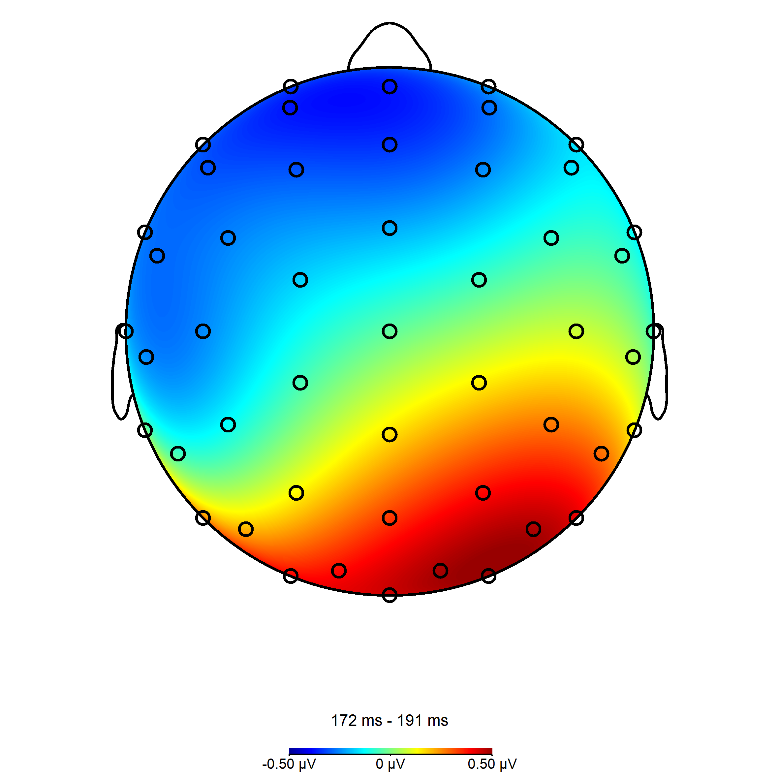 | 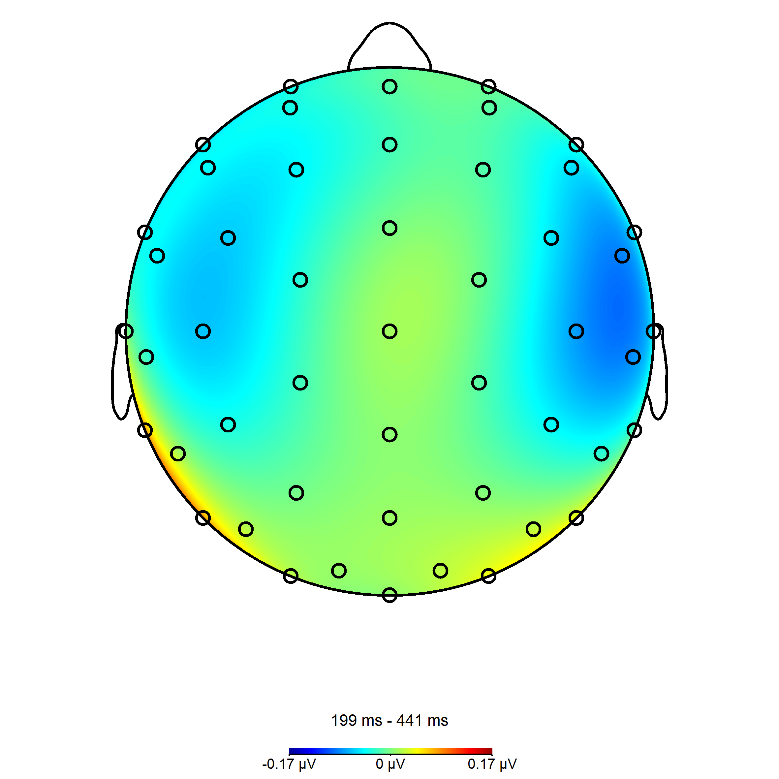 | 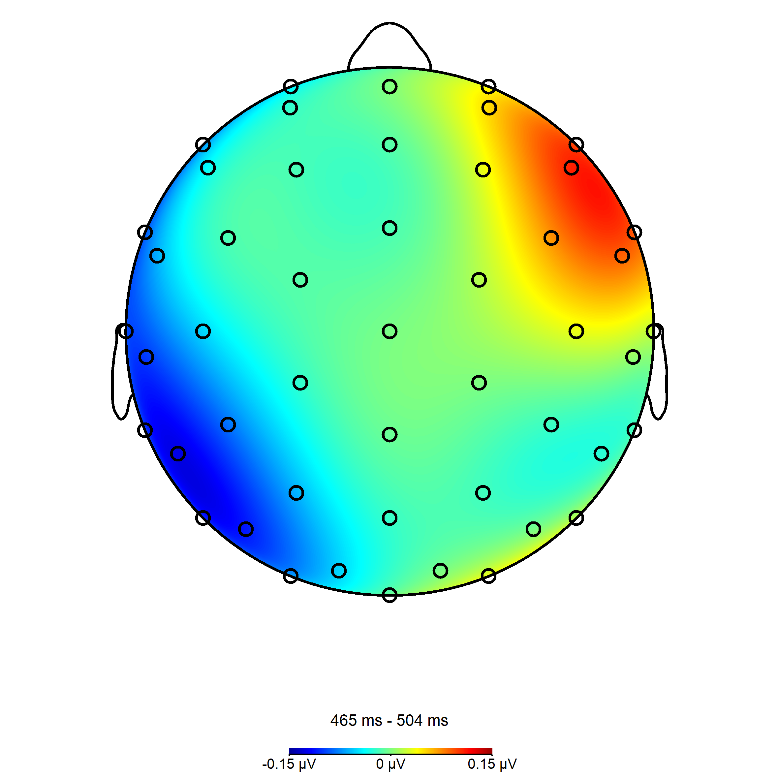 | 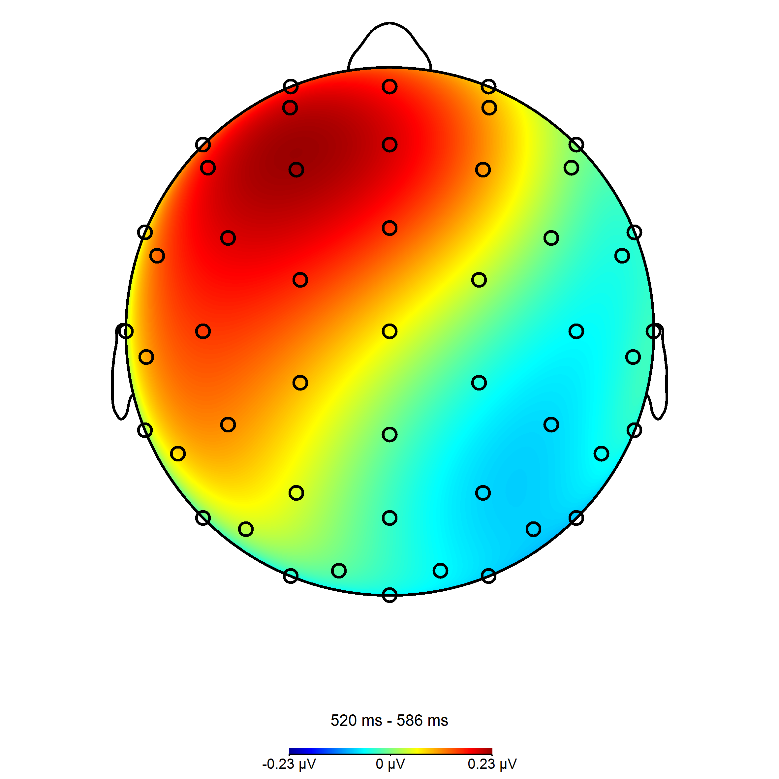 | 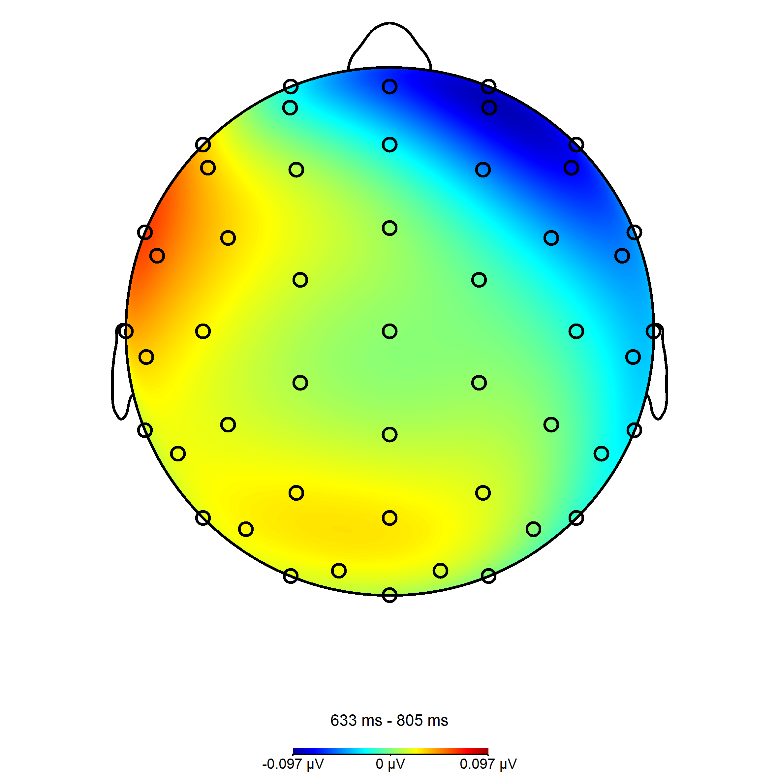 |
| 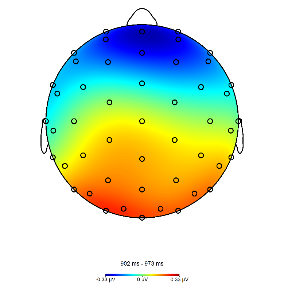 | 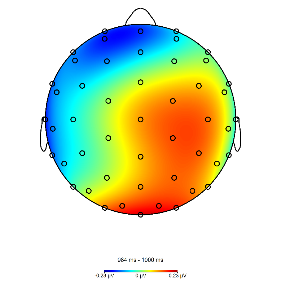 |  |  |  |
| 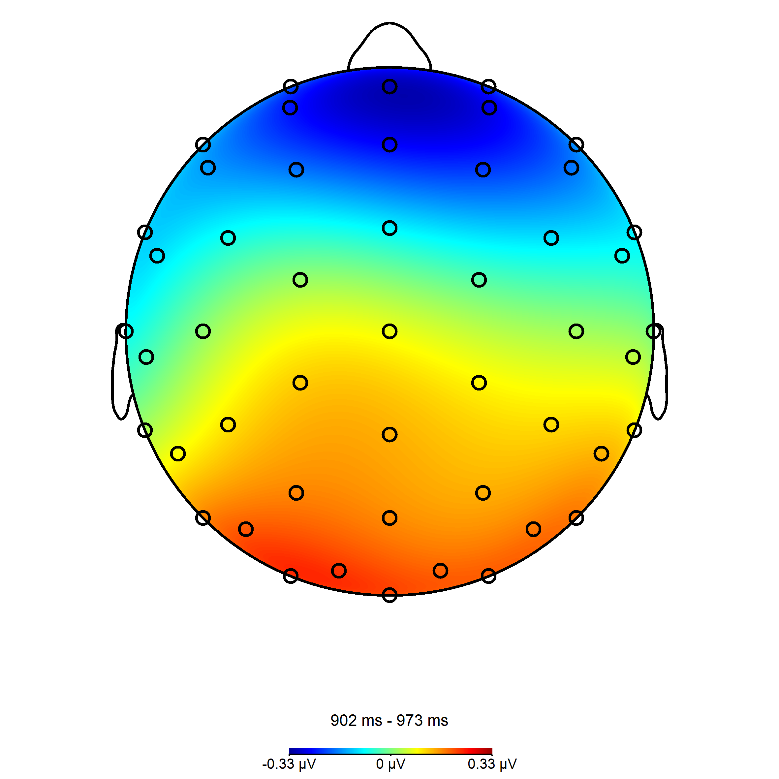 | 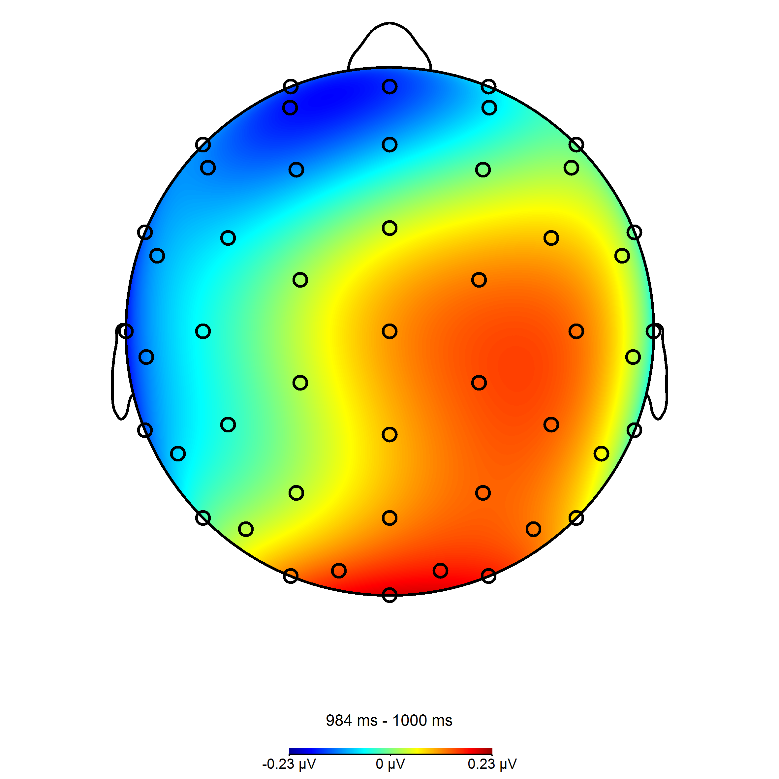 |  |  |  |
|  |  |  |  |  |

Audiobook group pre-appointment

| **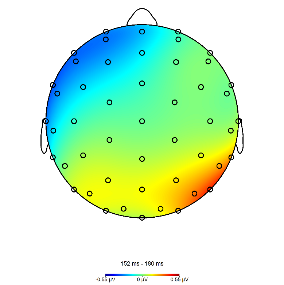** | **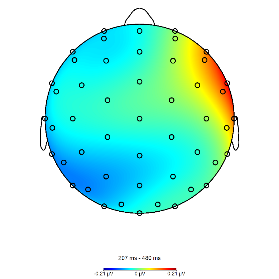** | **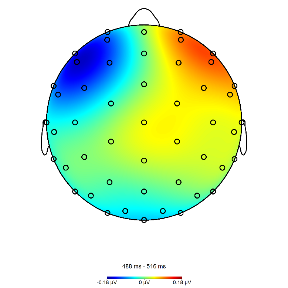** | **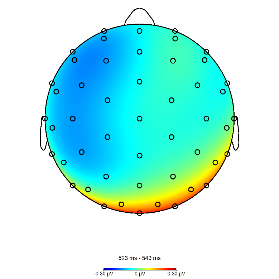** | **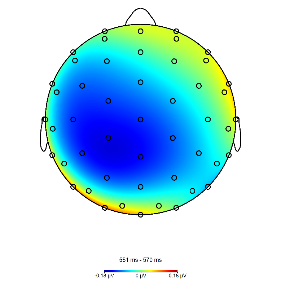** |
| --- | --- | --- | --- | --- |
| **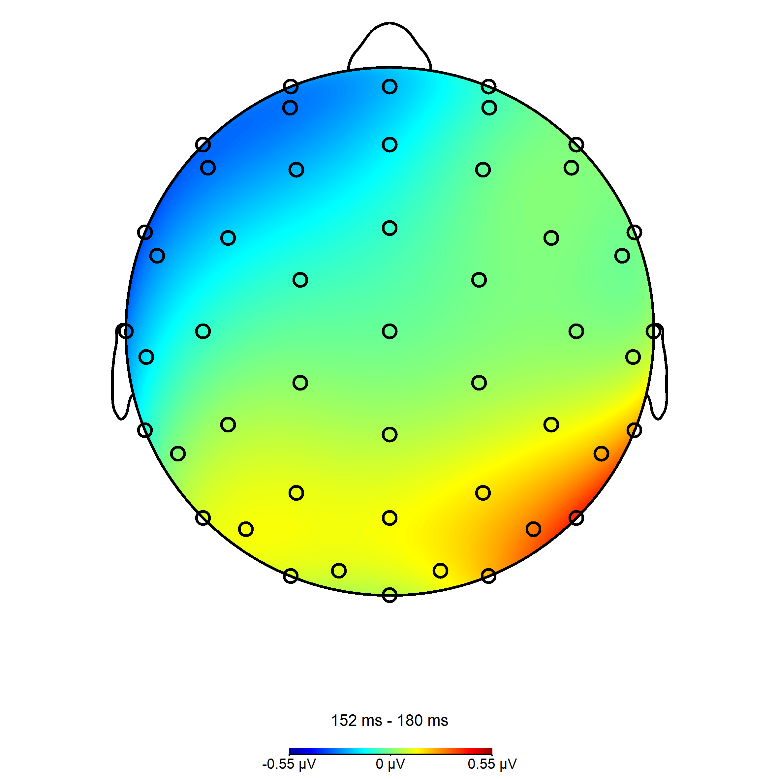** | **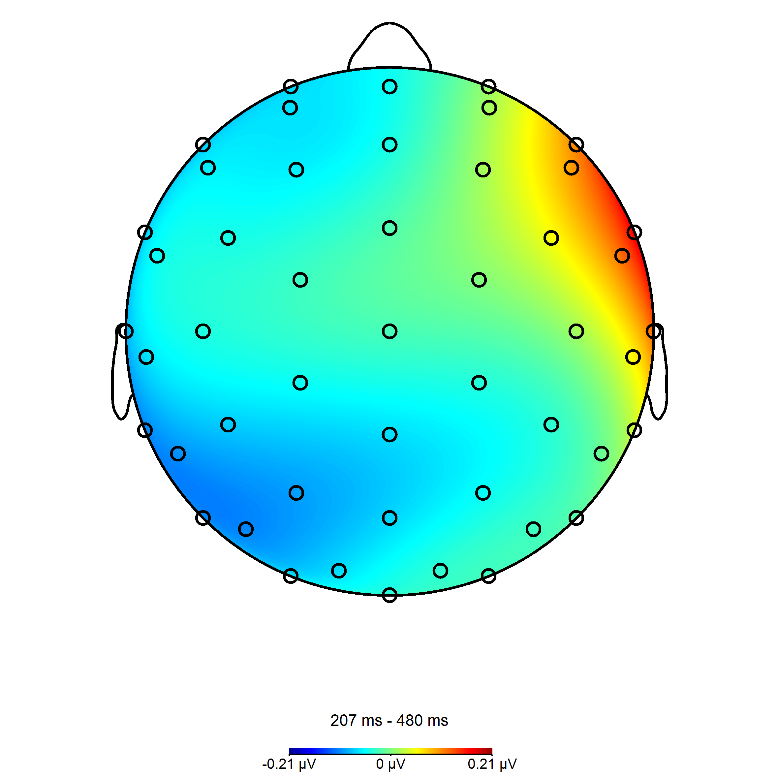** | **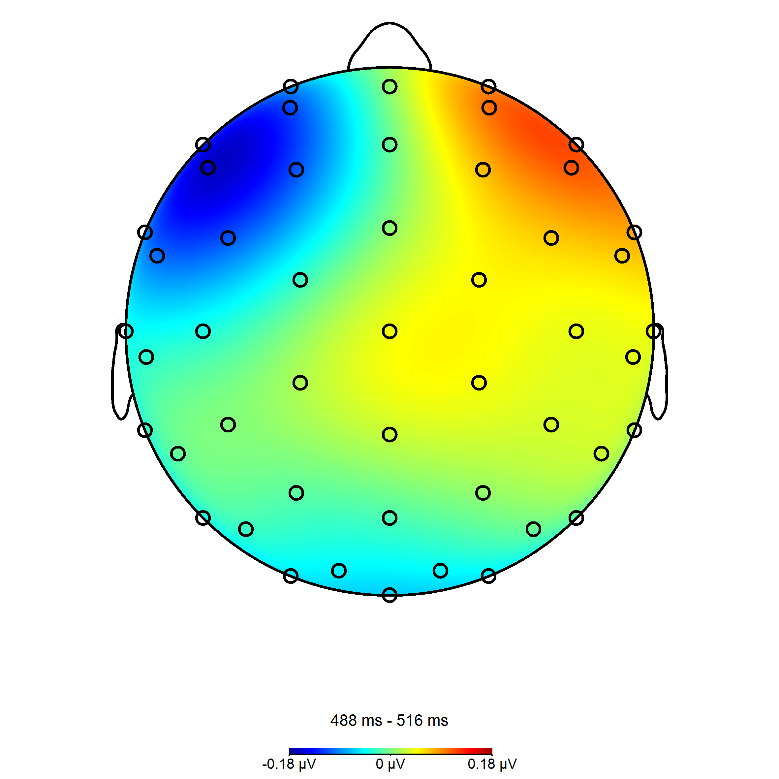** | **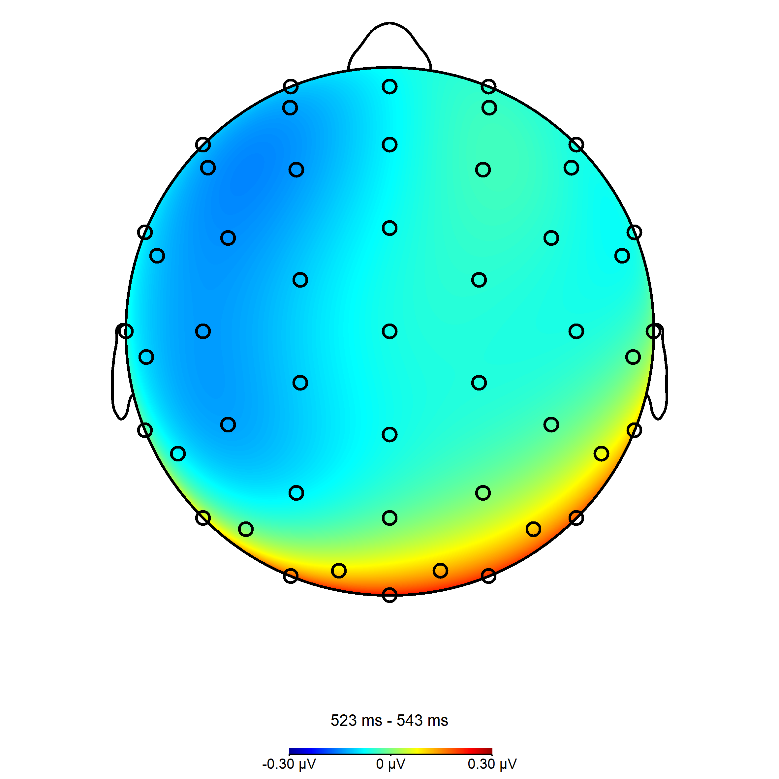** | **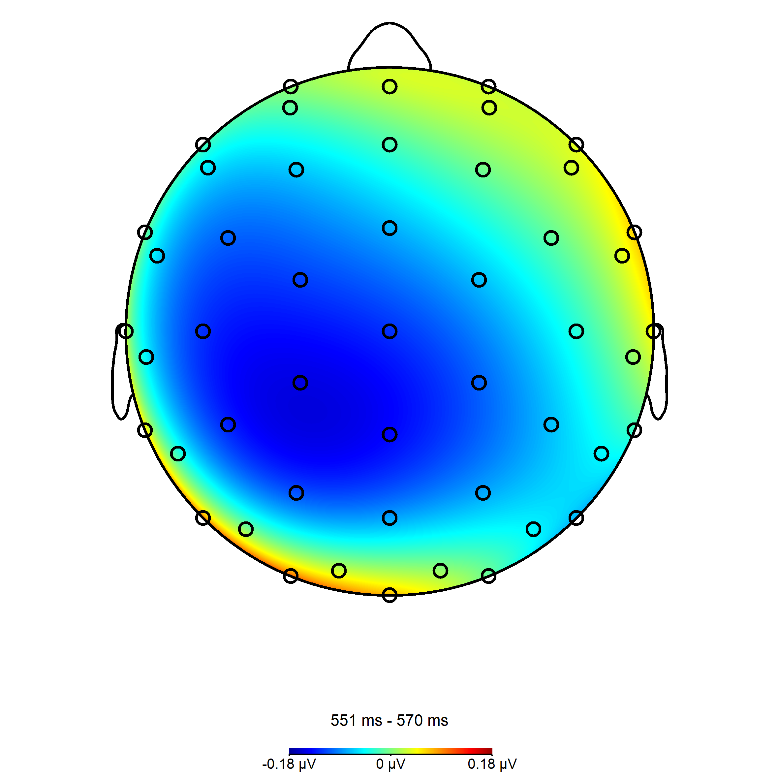** |
| **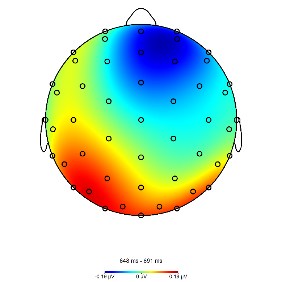** |  |  |  |  |
| **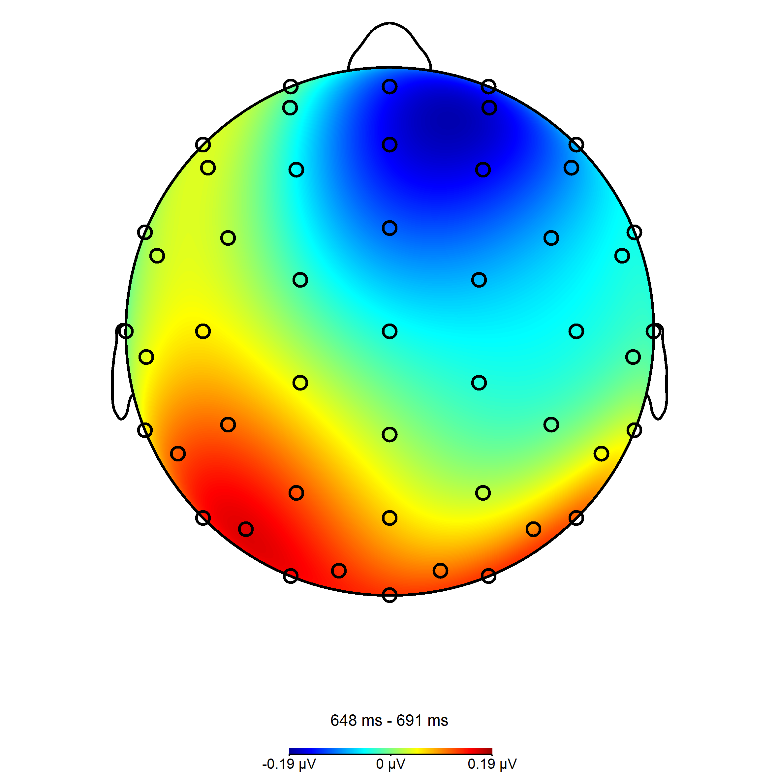** |  |  |  |  |

Audiobook group post-appointment

| **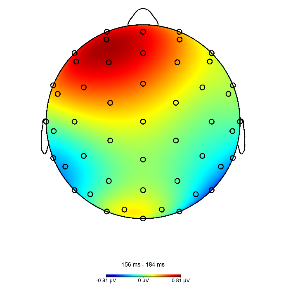** | **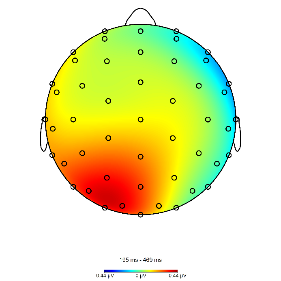** | **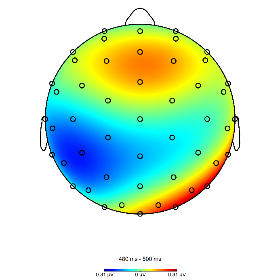** | **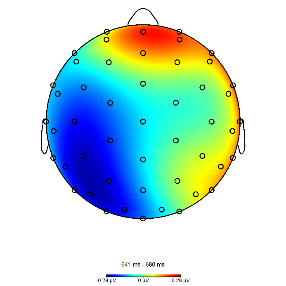** | **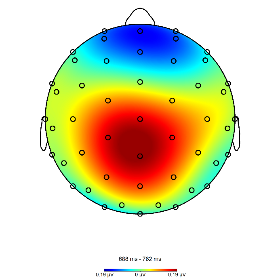** |
| --- | --- | --- | --- | --- |
| **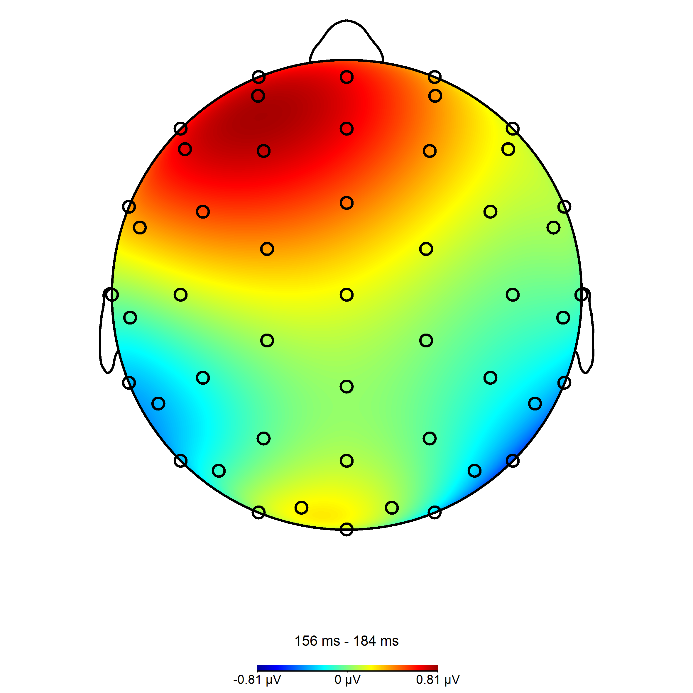** | **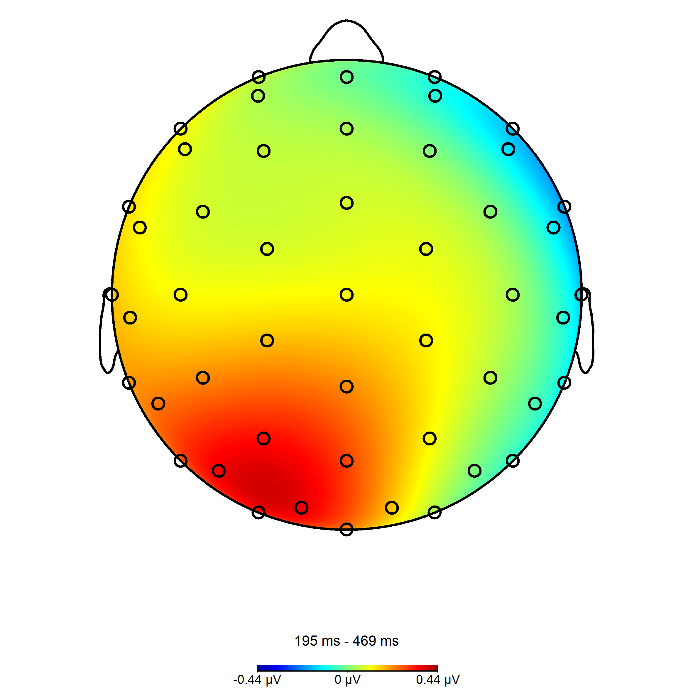** | **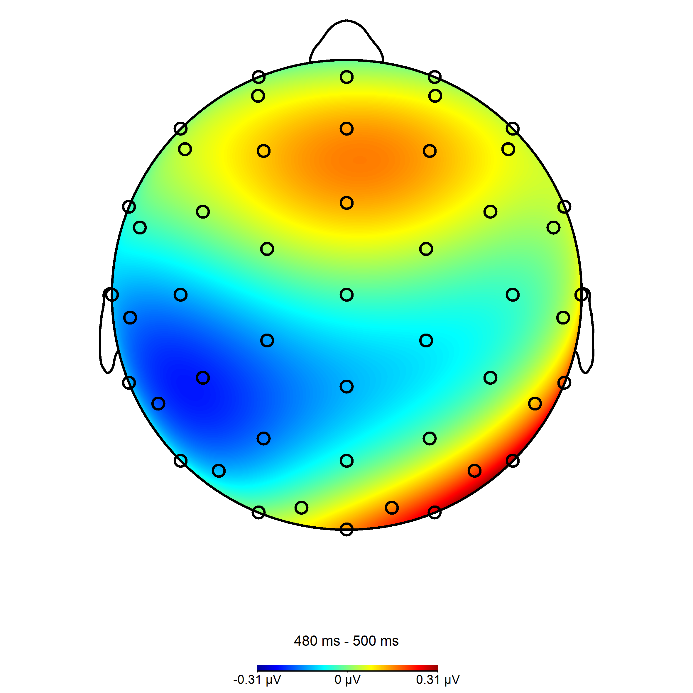** | **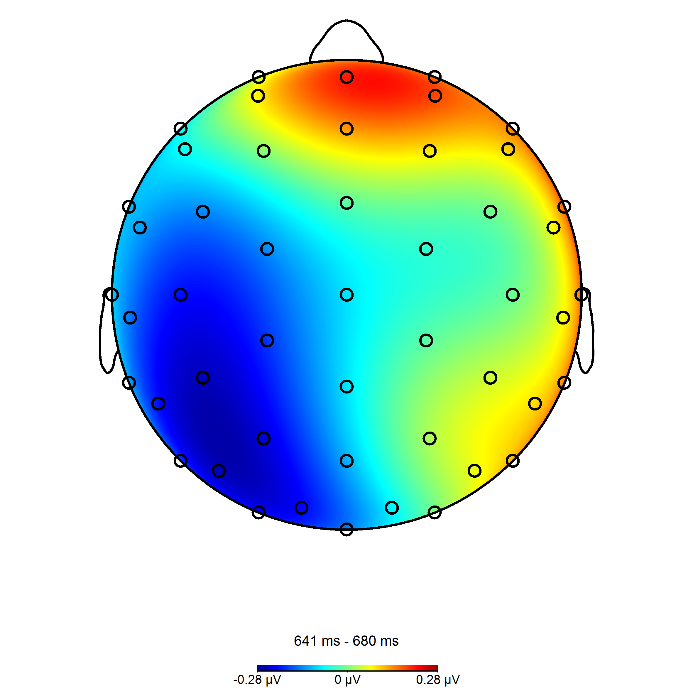** | **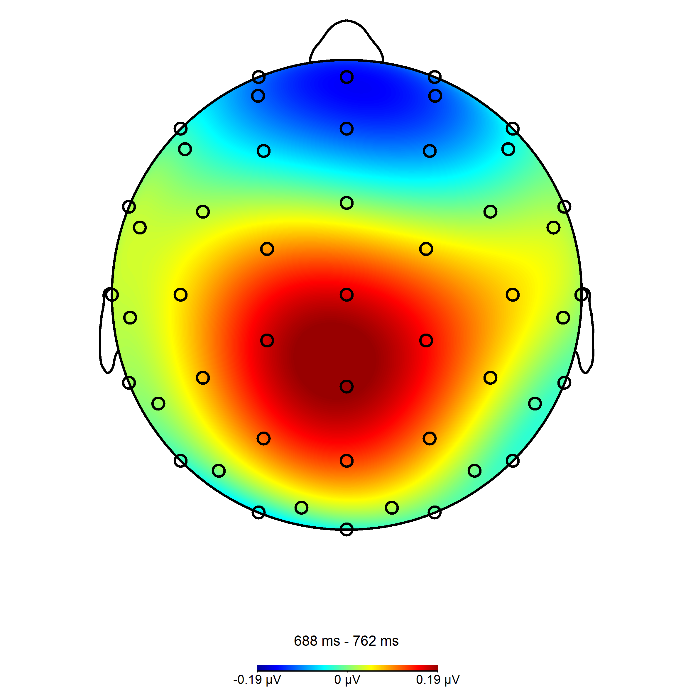** |
